# Widespread increasing vegetation sensitivity to soil moisture

**DOI:** 10.1038/s41467-022-31667-9

**Published:** 2022-07-08

**Authors:** Wantong Li, Mirco Migliavacca, Matthias Forkel, Jasper M. C. Denissen, Markus Reichstein, Hui Yang, Gregory Duveiller, Ulrich Weber, Rene Orth

**Affiliations:** 1 Department of Biogeochemical Integration, Max Planck Institute for Biogeochemistry, Jena, Germany; 2 Now at: European Commission, Joint Research Centre (JRC), Ispra, Italy; 3 Institute of Photogrammetry and Remote Sensing, Technische Universität Dresden, Dresden, Germany; 4 Hydrology and Quantitative Water Management Group, Wageningen University, Wageningen, The Netherlands; 5 Integrative Center for Biodiversity Research (iDIV), Leipzig, Germany

**Keywords:** Ecology, Ecology, Climate change, Carbon cycle

## Abstract

Global vegetation and associated ecosystem services critically depend on soil moisture availability which has decreased in many regions during the last three decades. While spatial patterns of vegetation sensitivity to global soil water have been recently investigated, long-term changes in vegetation sensitivity to soil water availability are still unclear. Here we assess global vegetation sensitivity to soil moisture during 1982-2017 by applying explainable machine learning with observation-based leaf area index (LAI) and hydro-climate anomaly data. We show that LAI sensitivity to soil moisture significantly increases in many semi-arid and arid regions. LAI sensitivity trends are associated with multiple hydro-climate and ecological variables, and strongest increasing trends occur in the most water-sensitive regions which additionally experience declining precipitation. State-of-the-art land surface models do not reproduce this increasing sensitivity as they misrepresent water-sensitive regions and sensitivity strength. Our sensitivity results imply an increasing ecosystem vulnerability to water availability which can lead to exacerbated reductions in vegetation carbon uptake under future intensified drought, consequently amplifying climate change.

## Abstract (web summary)

Water availability is a major control of vegetation dynamics and terrestrial carbon cycling. Here, the authors show that vegetation sensitivity to soil moisture has been increasing in the last 36 years, especially in (semi)arid areas, and that state-of-the-art land surface models fail to capture this trend.

## Introduction

Terrestrial vegetation is a crucial component in modulating the exchange of water, energy, and carbon between the land surface and the atmosphere^1–3^. At the same time, vegetation provides multiple essential ecosystem services such as food production and carbon uptake. The latter is critical for mitigating climate change by absorbing human-emitted CO_2_^4^. Vegetation requires sufficient energy and nutrients, and also soil moisture availability is essential, particularly in semi-arid regions^5,6^. As a result of ongoing climate change, soil moisture is declining in many regions as a consequence of decreased precipitation and higher evaporative water demand due to increased temperatures^7^. Related to this, the extent of regions where vegetation is dominantly controlled by the water supply has increased^8^, although increasing CO_2_ likely alleviates water stress by improving water use efficiency^9^. Yet, it remains unclear how climate change has affected the sensitivity of global vegetation to soil water availability, and if there are potential hotspot regions with high vegetation sensitivity to soil moisture where vegetation is particularly vulnerable to changes in soil moisture availability. Changes in vegetation water sensitivity relate to multiple processes: (i) soil drying and more frequent droughts can lead to increased sensitivity as water becomes more often limiting for plant activity^10^; (ii) plants can regulate water losses through their stomata (at the cost of decreased photosynthesis), which can prevent increased sensitivity to soil moisture through reduced water consumption^11^; and (iii) vegetation composition can affect ecosystem water sensitivity, for example, herbaceous and woody plants have different strategies to respond to soil dryness^12,13^. Quantifying and understanding the resulting sensitivity patterns and changes thereof are fundamental for inferring ecosystem vulnerability^3^, and have important implications for developing land surface models which can then contribute to more accurate predictions of the future terrestrial carbon sink and global climate^4,14,15^.

The increasing suite of Earth observations, including recent satellite-based vegetation and surface soil moisture products, now present the opportunity to assess the interplay between soil moisture and vegetation globally^1^. In fact, leaf area index (LAI) products and other vegetation indices related to vegetation greenness and productivity can represent long-term global vegetation growth dynamics^8,16–19^. They are routinely employed to study land-atmosphere interactions as they are sensitive to soil moisture dynamics, and can diagnose temporal sensitivity to environmental drivers thanks to their relatively higher signal-to-noise ratio than photosynthesis-related indicators such as sun-induced fluorescence^5^. Furthermore, LAI is readily available as a key prognostic variable from land surface models (LSMs)^20^. From a modeling perspective, a more accurate representation of LAI response to soil water stress requires the differentiation between soil layers, as near-surface soil moisture primarily controls soil evaporation and precipitation infiltration and co-varies more with atmospheric conditions^21^, while sub-surface soil moisture is a more relevant plant water source^5,22^. State-of-the-art soil moisture reanalyses cover multiple layers and allow for comprehensive analyses of the vegetation-water interplay by benefiting from satellite-observed surface soil moisture and in-situ multi-depth soil moisture measurements^23,24^.

Here we investigate the global sensitivity of LAI to soil moisture and sensitivity trends with observation-based data and model simulations between 1982 and 2017. For this purpose, we use an approach of explainable machine learning^25^ to study the relationship between LAI and soil moisture anomalies (de-trended and de-seasonalized; Methods: Data pre-processing), which can isolate the effect of soil moisture across layers on LAI from that of the other hydro-climate variables (i.e., air temperature, precipitation, vapor pressure deficit, solar radiation anomalies). Specifically, we employ the Shapley Additive Explanations (SHAP) method in random forest modeling to estimate the sensitivity of LAI to soil moisture (“overall sensitivity” hereafter; Methods: Overall sensitivity; Supplementary Fig. 1). Next to overall sensitivity, we estimate temporal variations of LAI sensitivity to soil moisture for 3-year-block data and analyze trends in temporal variations of sensitivity (Methods: Trends of sensitivity). We perform cross-validation for the random forest models for both the overall and the 3-year block sensitivities and disregard grid cells in the case of out-of-bag (OOB) R^2^ < 0. We use five long-term satellite-derived LAI datasets and ERA5-Land soil moisture reanalysis^24^, as well as modeled data from offline simulations from 9 TRENDY LSMs. To better understand LAI response to soil moisture across layers and fairly compare respective layers in observations and LSMs, we distinguish between near-surface (0-~10 cm) and sub-surface (~10-~100 cm) soil moisture for individual products and models (Supplementary Table 1). To validate the robustness and uncertainty of observation-based results, we use additional satellite-based vegetation indices (i.e., normalized difference vegetation index, NDVI, and kNDVI^26^) and alternative soil moisture reanalysis products (Methods: Observation-based data). In this study, we explore the sensitivity of global leaf area index to soil moisture by applying explainable machine learning to observation-based datasets. We show that this sensitivity is increasing in many regions of the globe during the last 3 decades, which is not reproduced by land surface models.

## Global patterns of LAI sensitivity to soil moisture

**Fig. 1:**
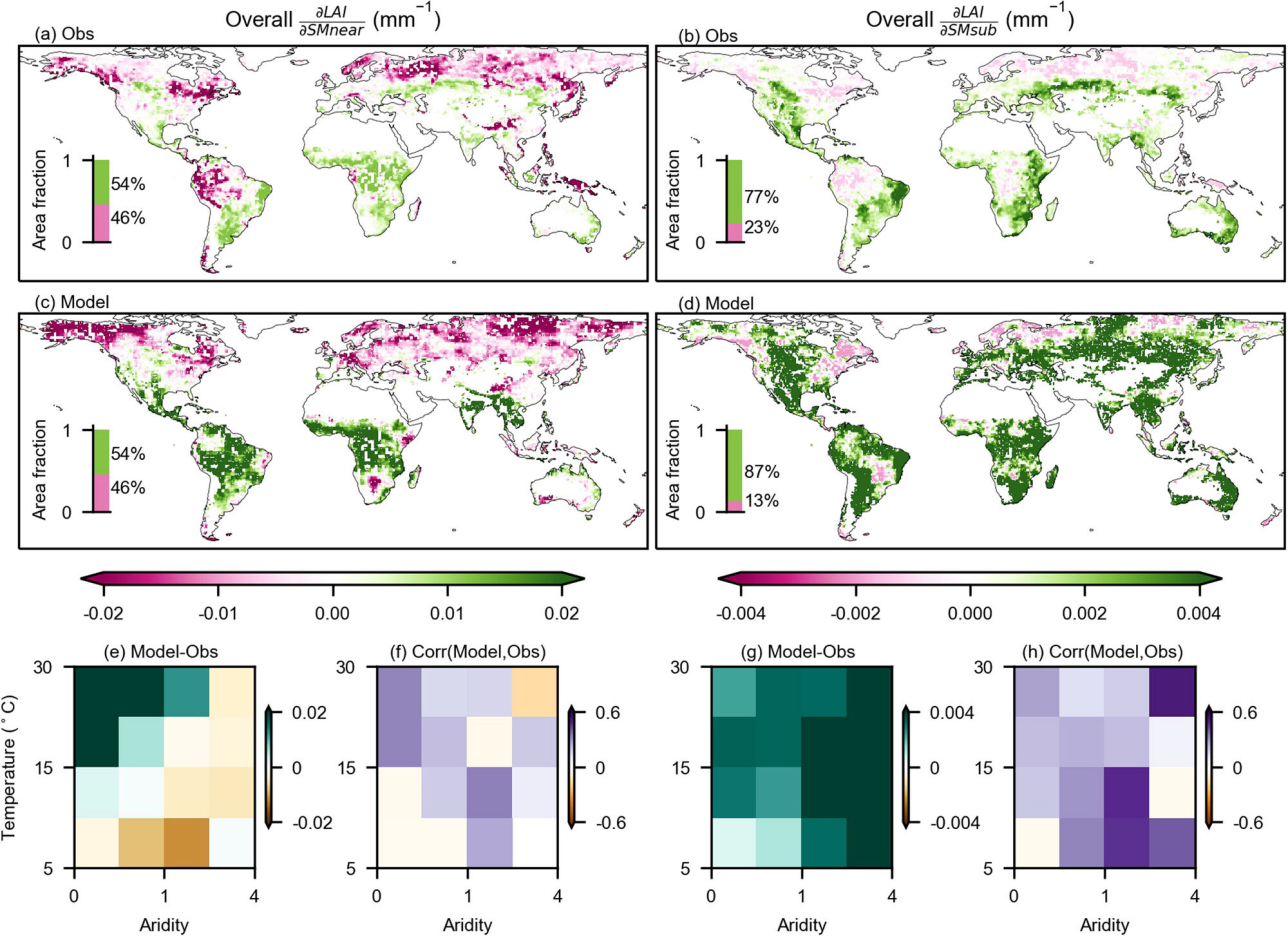
Global sensitivity of LAI to soil moisture in the period 1982-2017. **a**, **b** LAI sensitivity to near-surface ($\frac{\partial {LAI}}{\partial {SMnear}}$) and to sub-surface soil moisture ($\frac{\partial {LAI}}{\partial {SMsub}}$) from observations (Obs), given as respective ensemble means (Methods: Overall sensitivity). **c**, **d** Similar to **a**, **b** but for land surface models (Model). **e**, **g** Mean differences between observational and model results across climate regimes. **f**, **h** Spatial coherence between observational and model results inferred by correlation coefficients using sub-regional data across climate regimes. All panels apply the two-sided significance test at the *p* < 0.01 level as assessed with Theil-sen regressions for each grid cell, and grid cells which pass the significance test are colored according to the sensitivity values in **a**–**d**. To note that the results are a descriptive measure, as the field significance is not tested.

We analyze the overall sensitivity of LAI to soil moisture across the global land area where we disregard (i) irrigated and (ii) non-vegetated regions (Methods: Auxiliary data), as well as grid cells where (iii) the random forest model does not perform well (OOB R^2^ < 0) due to scarce vegetation activities or frequent human management (Supplementary Fig. 2). Observation-based results show that the area fraction of regions with positive LAI sensitivity to near-surface soil moisture is slightly higher than negative sensitivity (Fig. 1a). Significantly positive sensitivity (*p* < 0.01) indicates that increases in near-surface soil moisture enhance LAI dynamics. This is found in (semi-)arid regions such as southern North America, southern Eurasia, eastern and southern South America, Australia, South Africa and eastern Africa. Observed negative sensitivity in many boreal regions indicates that increased near-surface soil moisture tends to suppress LAI, potentially associated with the soil water excess such as waterlogging^27^. However, the negative LAI sensitivity to soil moisture is also likely caused by the confounding effects, because energy-related variables such as temperature and radiation have been identified as main controls on LAI in such regions, whereas soil moisture inversely covaries with these variables^2,3^. Focusing on sub-surface soil moisture, we find more widespread positive sensitivity (Fig. 1b), indicating a higher relevance of this moisture reservoir for LAI owing to the higher amount of plant roots exploiting this layer than the shallow near-surface layer^5,28^. Meanwhile, the magnitude of LAI sensitivity is higher for the near-surface soil moisture as in this layer there is a relatively high fraction of coarse roots, which allow for more efficient use of soil water for vegetation growth^29^. Main regional differences between LAI sensitivity to near-surface and sub-surface soil moisture are found in the African pantropics where temperature, and hence evaporative demand, is comparatively high, and precipitation is comparatively low^30^ such that typically water is evaporated by plants or from surfaces, stimulating vegetation growth before reaching the deeper soil.

The global patterns of LAI sensitivity to soil moisture in LSMs partly match the observation-based results (Fig. 1c-d). Differences exist for near-surface soil moisture where extra-tropical regions in the northern hemisphere and South Africa are not sufficiently reflected in the model results, and for sub-surface soil moisture where the models generally overestimate the positive sensitivity. When grouping the sensitivity results by the local long-term aridity and temperature conditions, we find that the observed LAI sensitivity changes predominantly along aridity gradients, while the modeled sensitivity tends to respond more strongly to temperature gradients (Supplementary Fig. 3; see aridity definition from Methods: Auxiliary data). Further comparing the observational with model results, we find overestimated (underestimated) LAI sensitivity in wet and hot (dry and cold) regions in the case of near-surface soil moisture (Fig. 1e). For sub-surface soil moisture, the strongest overestimation occurs in dry areas, while the bias is lower in wet areas (Fig. 1g). The spatial sensitivity patterns agree more with observational results in the case of sub-surface soil moisture (Fig. 1f, h).

## Non-linear relationships between LAI and soil moisture across space

**Fig. 2:**
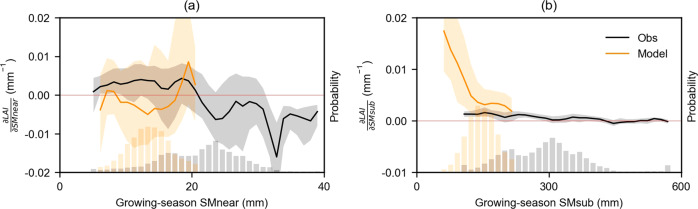
Response functions of global LAI sensitivity to soil moisture. **a** Response functions of LAI sensitivity ($\frac{\partial {LAI}}{\partial {SMnear}}$) to growing-season mean near-surface soil moisture (growing-season mean SMnear) from observations (Obs) and land surface models (Model). **b** Similar to **a** but for LAI sensitivity to sub-surface soil moisture ($\frac{\partial {LAI}}{\partial {SMsub}}$). In **a**, **b**, global grid cells with significant LAI sensitivities to soil moisture are included. Two-sided significance tests are done for each grid cell at the *p* < 0.01 level as assessed with Theil-sen regressions. The solid line and shaded areas show the median and interquartile ranges of LAI sensitivity. Probability distributions of near-surface soil moisture in observations and models are shown at the bottom of each plot. Results here are based on ensemble-product means, while results from individual products or models are presented in Supplementary Fig. 4.

Next, we analyze to which extent the differences between the results from models and observations are related to different response functions of LAI sensitivity to soil moisture. For this purpose, we build upon the relationships between significant LAI sensitivity to available amounts of growing-season soil moisture (Fig. 2; see Methods: Data pre-processing for growing-season definition). Observation-based results show that LAI sensitivity to soil moisture is typically high for dry conditions and decreases toward wetter conditions for both soil moisture layers. The results exhibit non-linear relationships (Fig. 2) in line with previous research using site measurements^6^. Model-based results are similar in the case of sub-surface soil moisture, even though with a more pronounced sensitivity increase towards dry soil moisture conditions^31^. Instead, for the sensitivity to near-surface soil moisture, we find considerable differences between observations and models. However, differences between individual models are substantial for both soil layers (Supplementary Fig. 4). The mismatch between models and observations and the divergence between models can be related to different representations of the processes occurring in the soil-vegetation continuum, roots profile and water potentials in models, which lead to differences in simulated soil moisture dynamics and soil-vegetation coupling^32,33^. Furthermore, soil moisture constraints on carbon allocation, leaf senescence, phenology, or photosynthesis^31,34^ are uncertain and differ between models, as well as the number and depths of soil layers, and their consideration for inferring vegetation water stress^34,35^. Vegetation water stress can also be related to atmospheric dryness (vapor pressure deficit) as well as soil dryness, while their relative roles are not fully understood and hence difficult to capture in models^15^. Nevertheless, the difference between the soil moisture amounts of reanalysis and LSMs should be interpreted with caution due to different soil and vegetation types employed in the reanalysis scheme versus that of LSMs^24,36^.

## Increasing LAI sensitivity to soil moisture

**Fig. 3:**
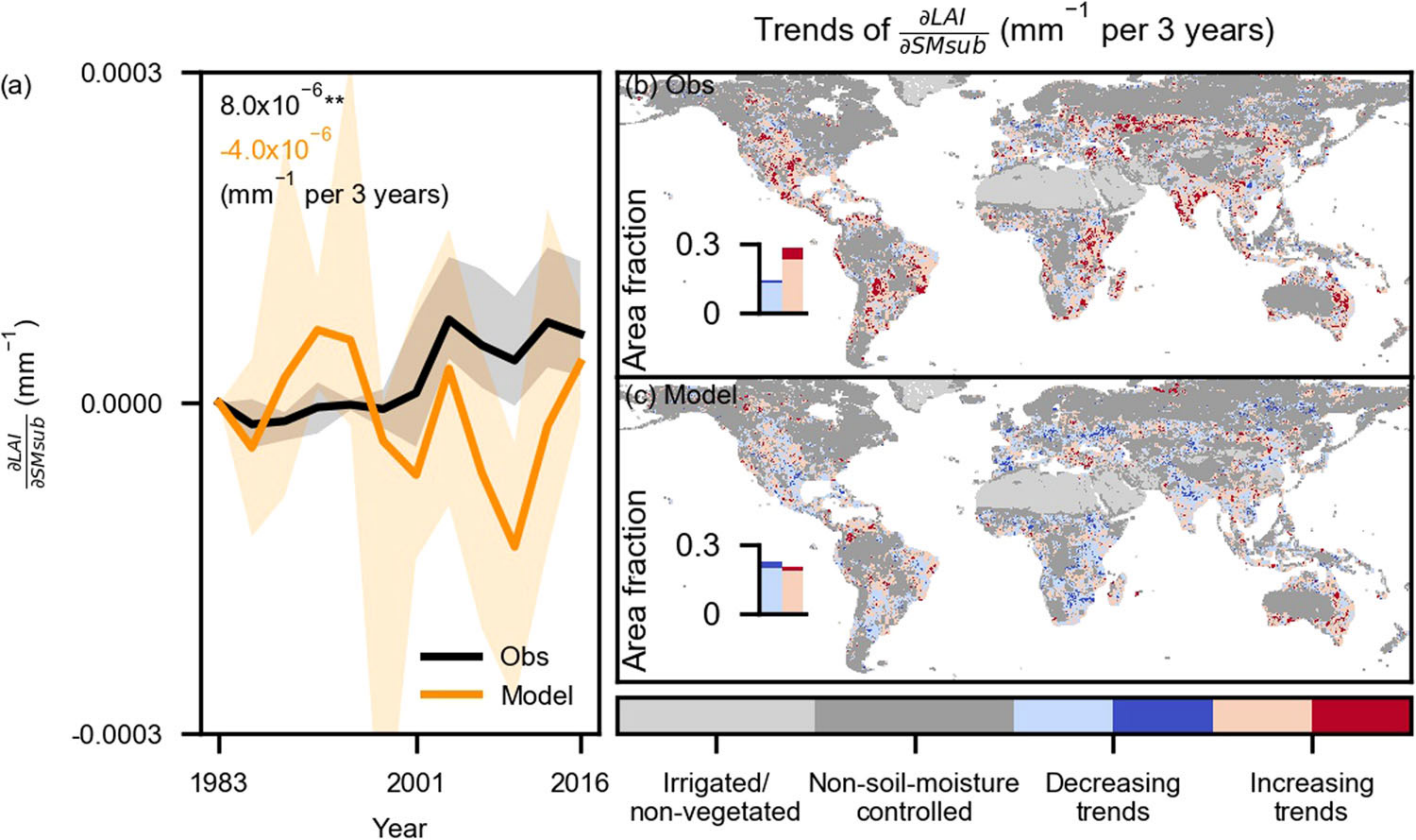
Trends of LAI sensitivity to sub-surface soil moisture. **a** Temporal variations of global mean LAI sensitivity to sub-surface soil moisture ($\frac{\partial {LAI}}{\partial {SMsub}}$) computed by 3-year blocks between 1982 and 2017. The y-axis denotes the change since 1982 in respective products or models. Solid lines denote the median results from ensemble observations (Obs) and land surface models (Model); Shaded areas denote interquartile ranges of LAI sensitivity from multiple LAI products and models; Text denotes slopes of trends; ** denotes passing the two-sided significance test as assessed with Mann-Kendall at *p* < 0.01 (Methods: Sensitivity trends). **b** Trends of LAI sensitivity to sub-surface soil moisture in observations and models using ensemble means. **c** Similar to **b** but for land surface models. Insets indicate the area fraction of decreasing and increasing trends within the global land area, excluding irrigated and non-vegetated regions. Light blue and red colors denote insignificant changes (*p* > =0.1); dark blue and red colors denote significant changes (*p* < 0.1). In **b**, **c**, two-sided significance tests are done for each grid cell at the *p* < 0.1 level as assessed with Mann-Kendall’s test. See Methods: Auxiliary data about the determination of irrigated/non-vegetated and non-soil-moisture controlled regions.

Moving beyond overall LAI sensitivity to soil moisture, we now analyze changes in the 3-year-block sensitivity to study their temporal variability from 1982 to 2017 (Fig. 3). For this purpose, we only consider soil moisture-controlled regions with significantly positive overall LAI sensitivity to soil moisture in observations and models to mitigate the influence of confounding effects (Fig. 1). We find significantly increasing trends (*p* < 0.01) for observed LAI sensitivity to sub-surface soil moisture after averaging global results (Fig. 3a). LAI sensitivity increases in ~30% of the study area and mainly occurs in central and southern North America, central Eurasia, India, Australia, eastern Africa, central and eastern South America (Fig. 3b). By contrast, LAI sensitivity decreases in ~15% of the study area, occurring in central South Africa, the African and Amazon extratropics, Central Europe, eastern and central Asia (Fig. 3b). Note that Fig.3 focuses on sub-surface soil moisture for simplicity while the observed LAI sensitivity to near-surface soil moisture is provided in Supplementary Fig. 5 also with significantly increasing trends globally. In addition, we validate the robustness of our methodology by (i) testing different thresholds for the random forest model performance as indicated by the OOB R^2^ (Supplementary Fig. 6) and (ii) repeating the analysis with 5-year blocks (Supplementary Fig. 7); we find similar results in both cases.

By contrast, there is no trend in LAI sensitivity to sub-surface soil moisture in TRENDY model simulations after averaging global results (Fig. 3a). The spread between the results of individual models is substantial, as indicated by the orange shading. We find similar extents of areas with regionally increasing and decreasing trends, respectively (Fig. 3c). Furthermore, we confirm similar results when focusing on respective soil moisture-controlled study areas inferred from either observations or models as defined from positive overall LAI sensitivity to soil moisture (Supplementary Fig. 8).

## Attribution of trends of LAI sensitivity to soil moisture

**Fig. 4:**
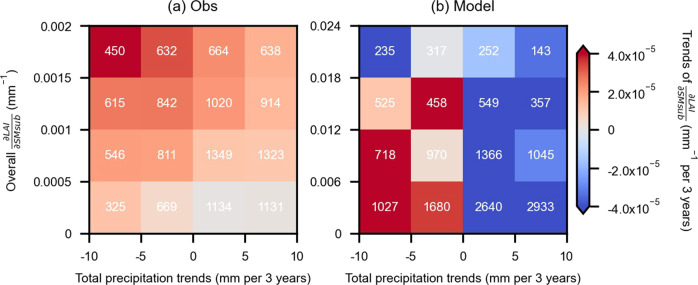
Trends of LAI sensitivity to sub-surface soil moisture $(Trends\,of\frac{\partial LAI}{\partial SMsub})$ grouped by precipitation trends and overall sensitivity $({{{{{\rm{Overall}}}}}}\,\frac{\partial LAI}{\partial SMsub})$. **a** Colors indicate median values of trends in LAI sensitivity to sub-surface soil moisture in observational ensemble means (Obs) grouped by precipitation trends and overall sensitivity; numbers of grid cells in each group are shown in white. **b** Similar as in **a** but for ensemble means of land surface models (Model).

We perform an attribution analysis to understand changes in LAI sensitivity to sub-surface soil moisture. We exclusively focus on sub-surface soil moisture in this context as LAI is often more strongly controlled by soil moisture in this layer, and the observed coupling between LAI and sub-surface soil moisture is captured relatively well by land surface models (Fig. 2). We explain sensitivity trends inferred from observations by relevant hydro-climate and ecological variables^8,10,12,13^ (Methods: Attribution analysis). We find that the observed spatial trend patterns are strongly related to (i) overall LAI sensitivity to sub-surface soil moisture and (ii) inter-annual precipitation trends (Supplementary Fig. 9). We group the results from Fig. 3b with respect to the identified main controls (Fig. 4a) and find that positive trends in LAI sensitivity to sub-surface soil moisture are strongest for regions with the largest overall sensitivity and the most substantial decrease in precipitation. Areas with high overall sensitivity include large ratios of grasses and shrubs which have strong roots’ hydraulic controls but weak stomatal regulation^11,12^. Small decreases in water supply tend to trigger drastic changes in LAI, reflecting a non-linear vegetation water response^37^. In a few regions, we find increasing LAI sensitivity despite weakly increasing precipitation trends, which relates to increased evaporative demand due to increasing temperatures. Moreover, our results suggest that deeper soil moisture or groundwater can generally not sustain a constant vegetation water sensitivity by compensating for precipitation decreases^38^. Soil moisture trends also play a role, even though they are less prominent than precipitation trends. This might be related to the higher observational uncertainty in soil moisture (trends) than precipitation (trends) owing to more indirect measurements and heterogeneous soils. Trends in energy-related variables (temperature, radiation, vapor pressure deficit), vegetation composition and changing composition (see Methods: Auxiliary data for non-tree cover) are of secondary importance.

We also evaluate the model-based spatial patterns of LAI sensitivity trends against the identified main controls (Fig. 4b). We find that precipitation decreases (increases) lead to increased (decreased) LAI sensitivity to soil moisture, consistent with the observation-based results. The role of overall LAI sensitivity in determining sensitivity trends in models is less clear than in the observations, suggesting that this key deficiency is behind the reason for the poor modeled trends in LAI sensitivity (Figs. 1 and 2).

Our observed results might be affected by three sources of uncertainty: (i) the satellite-based LAI products used in the analysis regarding their retrieval uncertainties and representativeness of the greenness and vegetation productivity, (ii) uncertainties in the soil moisture reanalysis products which rely on modeling assumptions, can be more pronounced for sub-surface soil moisture, as this layer does not have direct satellite data to assimilate, and (iii) artifacts in the long-term LAI time series derived from the Advanced very high-resolution radiometer (AVHRR) instrument. We address these uncertainties to assess the robustness of our main findings. First, our results show great consistency in the overall sensitivity (Supplementary Fig. 4) and sensitivity trends (indicated by the gray shading in Fig. 3a and Supplementary Fig. 5a) derived from ensemble long-term LAI products. The same analysis conducted on NDVI and kNDVI, alternative vegetation indices related to greenness and vegetation productivity, are consistent with LAI results (Supplementary Fig. 10). We also find consistency between our results and the ones obtained from Moderate Resolution Imaging Spectroradiometer (MODIS), which is characterized by higher quality but covering a shorter period than AVHRR (Supplementary Fig. 10). Second, our main results hold when employing different state-of-the-art soil moisture reanalysis products, which illustrate that our analysis is robust against different designs of model schemes (decreasing sensitivity patterns towards increasing soil moisture in Supplementary Fig. 4; increasing sensitivity trends in Supplementary Figs. 11 and 12). Moreover, we perform our analysis with soil moisture datasets that are independent of reanalysis products applying process-based models; One derived with machine learning-based extrapolation of in-situ measurements (SoMo.ml)^39^ shows similar global patterns of overall LAI sensitivity and global patterns of sensitivity trends obtained with ERA-Land for the same time period; And another one derived from satellite observations (ESA CCI soil moisture)^40^ confirms our main findings of significantly increasing global trends of LAI sensitivity to soil moisture (Supplementary Fig. 13). We further note that other water sources such as deep soil moisture, groundwater and bedrock water can also influence vegetation productivity^22^. We account for this by using satellite-observed total terrestrial water storage data from GRACE^41^, and find that sub-surface soil moisture reanalysis can largely capture the vegetation response to deep soil water in many regions, and LAI is even more related to the soil moisture reanalysis as it probably reflects more strongly the root-zone water availability (Supplementary Fig. 14). Third, issues such as sensor drifts in the AVHRR instrument can potentially introduce artifacts in LAI retrievals^18,19,42–44^. We largely account for potential biases and discrepancies between LAI products by using de-trended and de-seasonalized LAI data (Methods: Data pre-processing). Next to this, we also find that potentially spurious trends in the inter-annual variability of LAI cannot efficiently explain the observed patterns of LAI sensitivity trends (Supplementary Fig. 9). The consensus of most LAI products in terms of global patterns of increasing trends of LAI sensitivity to soil moisture further supports the robustness of our findings (Supplementary Fig. 15).

In conclusion, we show that the sensitivity of LAI to soil moisture has significantly increased in soil water-controlled regions during 1982-2017. This is driven primarily by decreasing water supply (i.e., precipitation) and modulated by LAI sensitivity to water availability. Our study illustrates that understanding changes in the soil moisture-vegetation interplay requires jointly considering changing climate^8,10^ and vegetation characteristics^12,13^ in the form of overall sensitivity. Our results are derived through explainable machine learning, which can essentially isolate the influence of soil moisture on LAI from that of other relevant drivers, and thereby goes beyond purely correlation-based analyses. Land surface models fail to capture the increasing LAI sensitivity to soil moisture, related to an inaccurate representation of overall LAI sensitivity in terms of spatial patterns and magnitude. Overall, the detected increasing vegetation sensitivity to soil moisture reflects enhanced ecosystem vulnerability to soil dryness. By identifying regions of strong and increasing sensitivity, our study highlights hotspot areas where decreasing soil moisture trends can induce severe impacts on vegetation and related carbon-climate feedbacks.

## Methods

### Observation-based data

We use five satellite-based LAI products that cover the period 1982-2017. This allows us to assess the robustness of our results with respect to the underlying differences in post-launch sensor calibration, corrections of orbital shifts and sensor degradation, as well as cloud and atmospheric corrections^44^. In particular, we employ the third generation Global Inventory Modeling and Mapping Studies LAI (GIMMS3g V1)^45^, the Land Long Term Data Record LAI (LTDR V5)^46^, the Global Land Surface Satellite LAI (GLASS V40)^47^, the Long-term Global Mapping LAI (GLOBMAP V3)^48^, and the GEOV2-AVHRR LAI products (https://www.theia-land.fr/wp-content/uploads/2020/11/THEIA-MU-44-0369-CNES-GEOV2-AVHRR-Product-User-Manual-V2.pdf)^49^. We note that the individual long-term LAI products used within our LAI ensemble account to different extents for biases such as sensor drifts, resulting in discrepancies in their estimated inter-annual trends and variability. We account for these discrepancies by removing long-term trends and mean seasonal cycles, as well as by confirming that potentially spurious trends in the inter-annual variability of LAI do not strongly influence our inferred LAI sensitivity trends. Furthermore, we find that LAI sensitivity trends from 4 out of 5 individual LAI products are significantly increasing which supports our main results. The exception is GLASS LAI which is known for its considerable differences compared with the other products in terms of trends and variability for pre-MODIS time period^43^, likely contributing to its divergent long-term changes in LAI sensitivity (Supplementary Fig. 15).

To further validate our results, we additionally use GIMMS3g v1 NDVI from 1982 to 2015 as an alternative vegetation index that does not rely on radiative transfer modeling, and similar products based on a different instrument, MODIS: MOD15A2H LAI and MOD13C2 NDVI from 2000 to 2017. Moreover, Kernel NDVI, which uses nonlinear generalization to better monitor vegetation productivity, is retrieved from MOD13C2 NDVI by following Camps-Valls et al., 2021^26^, using the recommended length-scale parameter of 0.5. MODIS products are selected with good quality flags, thereby ignoring low-quality data. We are not considering alternative vegetation indices or products derived from satellite observations such as sun-induced fluorescence and vegetation optical depth as they usually provide shorter records which are less suitable for long-term trend analysis. More specifically, (i) sun-induced fluorescence has a lower signal-to-noise ratio compared with the employed greenness-related indices^5^ and (ii) vegetation optical depth is more related to vegetation water content while we aim to focus on productivity and greenness^50^.

To keep consistency with LSMs-related analyses, we employ CRU-JRA v3.26 meteorological datasets, including temperature, surface downward solar radiation, vapor pressure deficit (VPD), total precipitation^51,52^ (https://www.dropbox.com/sh/nlwz4n4r2k02ovb/AAC7BqTjS8fe4CR2IWWAfnRMa?dl=0). To analyze the water constraint of observational LAI, we use soil moisture from the ERA5-Land reanalysis^24^ where we use layer 1 (0-7 cm depth) as near-surface soil moisture and the weighted mean of layers 2 (7-28 cm) and 3 (28-100 cm) as sub-surface soil moisture. This state-of-the-art reanalysis data has been successfully applied to understand vegetation responses to water availability^8,53,54^. Soil moisture estimates from deeper layers in ERA5-Land are less constrained by observations, which is also true for its predecessor ERA-Interim/Land. In fact, the latter soil moisture products have been successfully evaluated many times against in-situ observations from global hydrology networks and has also been widely compared with other soil moisture reanalyses^55–60^.

We consider four additional global soil moisture products to validate our results: (i) the Modern-Era Retrospective analysis for Research and Applications-Version 2 (MERRA-2, 1982-2017)^61^, (ii) the Global Land Evaporation Amsterdam Model (GLEAM v3a, 1982-2017)^62^; (iii) a machine-learning-based product trained with multi-layer in-situ measurements (SoMo.ml, 2000-2017)^39^; and (iv) the satellite-derived ESA CCI surface soil moisture (1982-2017)^40^. Since the validation of the newly published ERA5-Land soil moisture reanalysis has only recently been done using in-situ measurements during 2010-2018^24^; the usefulness of this product for longer-term analyses as in our study can be deduced from successful long-term validations of related products from the European Centre for Medium-Range Weather Forecasts (ECMWF)^55,56^ which are based on the same land surface model and similar parameterisations. Additionally, the GLEAM soil moisture reanalysis which we also use is validated against over one thousand in-situ measurements during 1980-2015^62^. The global soil moisture product SoMo.ml takes advantage of large numbers of long-term in-situ measurements with machine learning algorithms, such that it can learn the relationship between meteorological input data and resulting soil moisture dynamics. Applying these machine learning algorithms in data sparse regions to obtain a global gridded soil moisture product is a way to transfer knowledge between data-rich and data-poor regions. SoMo.ml is limited by its time coverage from 2000 to 2017 but supports our main results by confirming the global patterns of overall LAI sensitivity and the global patterns of sensitivity trends obtained with ERA5-Land for the same time period (Supplementary Fig. 13). Similar to the results obtained with the ERA5-Land reanalysis soil moisture, ESA CCI yields an increasing trends of LAI sensitivity to soil moisture (Supplementary Fig. 13). The trend based on ESA CCI data seems more pronounced, even though the absolute values of the LAI sensitivity can not be compared due to different soil moisture units. Given that similar LAI sensitivities to soil moisture are derived with multiple independent soil moisture products, we note that our findings are robust despite the variability between existing soil moisture products.

To account for the potential vegetation responses to deep water sources, we additionally study LAI sensitivity to total water storage data from GRACE^41^. GRACE measures the anomalies of the Earth’s gravity field that can inform relative changes in the land water storage. Due to its limited observed time period 2003-2017, we only compare the overall LAI sensitivity to total water storage and to sub-surface soil moisture for this time period.

Soil layers considered for near-surface and sub-surface soil moisture are listed in Supplementary Table 1. The unit of all considered soil moisture data is converted from m^3^/m^3^ to mm using the respective layer depths to be consistent with the soil moisture unit used in land surface models; however, this conversion could not be applied for ESA CCI soil moisture since the observation depth differs in time and space depending on the penetration depth of the microwave frequency and the soil wetness, so that the absolute values of LAI sensitivity to soil moisture are not comparable with that of the other products^41^.

### Model data

To illustrate the performance of LSMs with the aspect of vegetation-soil moisture interplay, we simulate monthly LAI and multi-layer soil moisture during 1982-2017 using 9 models from the TRENDY v7. These models are ISAM, LPX-Bern, CLM5.0, JSBACH, JULES, ORCHIDEE-CNP, LPJ-GUESS, VISIT, and CABLE-POP. Factorial simulations are derived from Scenario 3, which include variable CO2, climate, and land-use changes. To thoroughly study interactions within LSMs and to fairly compare model results with observations, we use the same climate forcing CRU-JRA v2.0 datasets^51,52^ (https://catalogue.ceda.ac.uk/uuid/7f785c0e80aa4df2b39d068ce7351bbb) including temperature, surface downward solar radiation (solar radiation), vapor pressure deficit (VPD; derived from temperature and relative humidity), total precipitation in all observational and LSMs-related analyses. All the data are derived by following the TRENDY-v7 protocol^63,64^. We manually aggregate the multi-layer soil moisture into near- and sub-surface soil moisture. The near-surface soil moisture in the article refers to the layer approaching 10 cm, while sub-surface soil moisture refers to the layer approaching 100 cm (Supplementary Table 1). For LSMs including only 2 layers we leave it as it is. We note that also the observation-based results are subject to uncertainty, in particular related to the soil moisture reanalysis data which can potentially be degraded by imperfect soil and vegetation type representations in the land surface model underlying the reanalysis. But the sub-surface soil moisture reanalysis can benefit from the assimilation of other data streams such as precipitation and radiation, as well as the satellite-based near-surface soil moisture assimilation helps to simulate sub-surface soil moisture through the infiltration process and mitigate model errors.

### Auxiliary data

The VCF5KYR fraction of vegetation cover data includes three types of land cover and land use fraction: tree cover, non-tree cover, and bare ground^65^. The global study area is defined by the total vegetation cover (sum of tree and non-tree cover) ≥ 5% using 1982 -2016 averages and by the fraction of irrigation cover ≤ 10% (Data are collected around 2005; http://www.fao.org/aquastat/en/geospatial-information/global-maps-irrigated-areas/latest-version/)^66^. Irrigated or non-vegetated regions are the remaining land areas except the global study area. Non-soil-moisture controlled regions are defined as areas where LAI is not positively sensitive to soil moisture in observations and models. The area fraction in global maps is the number of grid cells weighted by the actual areas according to the geographic coordinates. The VCF5KYR fraction of vegetation cover data is also used to distinguish non-tree cover fraction by the ratio between non-tree cover (e.g., grasses and shrubs) and total vegetation cover as one of the ecological variables reflecting vegetation composition in the attribution analysis.

Climate regimes are applied to analyze global patterns of overall LAI sensitivity, and defined by the aridity index and long-term mean temperature data using ERA5-Land data. The aridity index is calculated as the ratio of the long-term mean net radiation and unit-converted precipitation^67^. Aridity values higher than 1 denote semi-arid regions or dry conditions.

### Data pre-processing

We provide a flowchart of data-processing and the sensitivity analysis in Supplementary Fig. 1. All observational and LSMs data are aggregated to monthly temporal resolution, and 0.5°x0.5˚ spatial resolution, including that a few models are upscaled regarding spatial resolutions. In all experiments and all vegetation and hydro-climate variables, we select growing-season data by temperature>5˚C and ensemble LAI means from the original signals>0.5 to keep a temporal consistency, whereas additionally negative values of vegetation indices are filtered out. Seasonality and long-term trends are removed to obtain the anomaly of every single vegetation and hydro-climate variable by subtracting long-term mean monthly signals and by subtracting a locally weighted smoothing filter^68^ with a smoothing span of 0.4, respectively. In this way, we exclude long-term common trends derived by changes in the equilibrium state, such as long-term successional cycles or human overgrazing. We also largely exclude biases from multi-sensor shifts and focus specifically on short-term vegetation responses to soil moisture anomalies.

### Overall sensitivity

Note that overall sensitivity, temporal variations of sensitivity and sensitivity trends are first computed for each observational product (or land surface model) and then averaged across products to obtain more robust multi-product estimates.

We use explainable machine learning (SHapley Additive exPlanations) to study LAI sensitivity to soil moisture availability by disentangling the contribution of (i) near-surface soil moisture to LAI anomalies from the influence of other variables including sub-surface soil moisture and (ii) similarly of sub-surface soil moisture from the influence of other variables including near-surface soil moisture. For this purpose, we first train Random forests models and then apply SHapley Additive exPlanations (SHAP) to isolate the marginal contributions of each predictor on the target variable. Random forests are one of the data-driven machine learning algorithms based on a bootstrap aggregating strategy for improving results stability, and it requires no statistical assumptions on predictors and target variables using sufficient numbers of data^69^.

For each LAI product from observational data or LSMs, we treat the LAI anomaly as the target variable and corresponding hydro-climate anomalies as predictors by a common hyperparameter setting optimized by grid-cell level tests (numbers of estimators: 100; maximum features: 30%; random state: 42). We collect all predictors and target data during 1982-2017 from one grid cell and the surrounding grid cells (3×3 shape) to train a model for the core grid cell if more than 50 data points are included. We remove grid cells that have model performance worse than the mean of training data itself using cross-validation out-of-bag score (OOB R^2^ > 0). We note that the rather low threshold (OOB R^2^ > 0) is selected because of a typically significantly decreased model performance in predicting global vegetation productivity for anomalies compared to time series that include the mean seasonal cycles^5,70^, while it can still be efficiently used to study relationships between predictor variables and targets. Regions with R^2^ < 0 are mostly associated with very low LAI variability or frequent human management (Supplementary Fig. 2), and the increasing thresholds of OOB R^2^ do not affect our main conclusions (Supplementary Fig. 6).

For one trained model, we apply SHAP dependence method to isolate marginal contributions of near-surface (or sub-surface) soil moisture on the LAI anomaly^71^. We define overall LAI sensitivity as the slope estimated from Theil-sen regression between SHAP dependence for LAI and near-surface (or sub-surface) soil moisture anomalies by assuming that grid cell-level interaction between LAI and soil moisture is nearly linear^5^. Overall sensitivity is first computed for each observational LAI product or land surface model before averaging the results to yield more robust multi-product estimates.

Because the sensitivity is inferred by a linear regression, it should not be expected to represent the full interactions between vegetation and soil moisture per grid cell. This method combines the advantages of bootstrap aggregating and non-distribution-assumption by random forest modeling, as well as advantages of global interpretations being consistent with the local explanations in the SHAP algorithm^5,71,72^, hence strengthening the robustness of the results than using traditional statistical methods.

### Trends of sensitivity

Grid cells with negative overall sensitivity or non-significant (p > =0.1) results are defined as non-soil-moisture controlled regions, meaning that energy-related variables such as radiation could dominantly control vegetation growth, and the detected dependence on water is likely due to confounding effects. Therefore, we remove these grid cells in the first place of studying changes in vegetation-water relationships. To address temporal variations of LAI sensitivity to near- and sub-surface soil moisture, respectively, we split the data from the entire 1982-2017 analysis period into twelve 3-year blocks (1982-1984, 1985-1987, …, 2015-2017). We train models independently again by 3 × 3 data points for each core grid cell if more than 15 data points are included, and infer temporal sensitivity by SHAP and Theil-sen regression by further assuming that grid cell-level interaction between LAI and soil moisture within 3-year blocks is nearly linear. We remove grid cells that have model performance worse than the mean of training data itself using cross-validation out-of-bag score and show non-significant (*p* > =0.1) results from Theil-sen regression.

We use the Mann-Kendall’s test to detect the trends of changes in LAI sensitivity which does not require data with normal distribution^73^. To confirm the 3-year split would not bias results, we also detect trends of 5-year-block sensitivity and find no significant differences (Supplementary Fig. 7).

### Attribution analysis

To better understand trends of LAI sensitivity to sub-surface soil moisture, we again apply random forests and the SHAP attribution method to predict trends of LAI sensitivity to sub-surface soil moisture^69,71^. We focus on sub-surface soil moisture in this context as LAI is often more strongly controlled by this layer. Note that at the same time, near-surface soil moisture is still included as a predictor in the random forest model to infer LAI sensitivity to sub-surface soil moisture, but the respective sensitivity to near-surface soil moisture is not evaluated. We treat sensitivity trends as the target variable, and multiple hydro-climate and ecological factors from growing seasons as predictors to train a model using global grid cells, and then we employ SHAP values to quantify marginal contributions of each single factor on sensitivity trends and then rank global-relevant variable importance by SHAP importance algorithm (absolute weighted averaged marginal contributions from each predictor variable). After identifying the dominant factors for sensitivity trends (Supplementary Fig. 9), we present combined impacts from the top two important variables which are precipitation trends and overall sensitivity and elucidate potential mechanisms across grouped ecosystems (Fig. 4).

### Reporting summary

Further information on research design is available in the Nature Research Reporting Summary linked to this article.

## Supplementary information

Supplementary Information

Reporting Summary

## Data Availability

Five long-term LAI products GIMMS3g V1, LTDR V5, GLASS V40, GLOBMAP V3 and GEOV2-AVHRR are available at http://sites.bu.edu/cliveg/datacodes/, https://ladsweb.modaps.eosdis.nasa.gov/, http://www.glass.umd.edu/, https://zenodo.org/record/4700264#.YRPUpNMzZlc, and https://www.theia-land.fr/en/geov2-avhrr-monitoring-changes-in-vegetation-on-a-global-scale-over-the-last-38-years/, respectively. GEOV2-AVHRR was produced and distributed by CNES based on the algorithm developed by CREAF and INRAE in the framework of the Theia Land Data Centre. GIMMS3g v1 NDVI, MOD15A2H LAI and MOD13C2 NDVI are available at https://lpdaac.usgs.gov/products. ERA5-Land climate and soil moisture reanalysis datasets are available at https://www.ecmwf.int/en/era5-land. GLEAM soil moisture reanalysis is from https://www.gleam.eu/. MERRA-2 soil moisture reanalysis is from https://gmao.gsfc.nasa.gov/reanalysis/MERRA-2/FAQ/. Fractional vegetation cover is from the AVHRR vegetation continuous fields products (VCF5KYR, https://lpdaac.usgs.gov/products/vcf5kyrv001/). Simulations from TRENDY land surface models are available on request to S.S. (s.a.sitch@exeter.ac.uk) and P.F. (p.friedlingstein@exeter.ac.uk).

## Code availability

The codes required for reproducing the results and figures in the main text have been deposited at 10.5281/zenodo.6554966, as well as the data to run the codes are available at 10.5281/zenodo.6445997.
