# Supplementary material for: Widespread increasing vegetation sensitivity to soil moisture
Source: Nat Commun. 2022 Jul 8;13:3959. doi: 10.1038/s41467-022-31667-9 (PMC9270344; doi:10.1038/s41467-022-31667-9)
Supplement: Supplementary file 1 — Supplementary Information [file 41467_2022_31667_MOESM1_ESM.pdf]

Supplementary information:

Supplementary Table 1: Depths and layers for aggregated near- and sub-surface soil moisture from observation-based products and land surface models. *LPX-Bern applies the two-bucket method which does not define specific depths of two soil layers; *LPJ-GUESS, VISIT and CABLE-POP are not used in averaging results of LSMs-based LAI sensitivity to near-surface soil moisture, due to incompatible value ranges of near-surface soil moisture amount with the other models (Supplementary Fig. 3).

		Near-surface soil moisture	Sub-surface soil moisture
Reanalyses	ERA5	Layer 1: 0-7 cm	Layer 2-3: 7-100 cm
	GLEAM	Layer 1: 0-10 cm	Layer 2: 10-100 cm
	MERRA-2	Layer 1: 0-5 cm	Layer 2: 5-100 cm
Observation-based	SoMo.ml	Layer 1: 0-10 cm	Layer 2-3: 10-50 cm
Land surface models	ISAM	Layer 1-3: 0-9 cm	Layer 4-7: 9-82.56 cm
	LPX-Bern	Layer 1-4: first bucket*	Layer 5-8: second bucket*
	CLM5.0	Layer 1-2: 0-6 cm	Layer 3-8: 6-92 cm
	JSBACH	Layer 1: 0-6.5 cm	Layer 2-3: 6.5-122.5 cm
	JULES	Layer 1: 0-10 cm	Layer 2-3: 10-100 cm
	ORCHIDEE-CNP	Layer 1-6: 0-9 cm	Layer 7-10: 9-150 cm
	LPJ-GUESS	Layer 1: 0-50 cm*	Layer 2: 50-150 cm
	VISIT	Layer 1: 0-30 cm*	Layer 2: 30 cm-200 cm
	CABLE-POP	Layer 1-2: 0-8 cm*	Layer 3-4: 8-64.3 cm

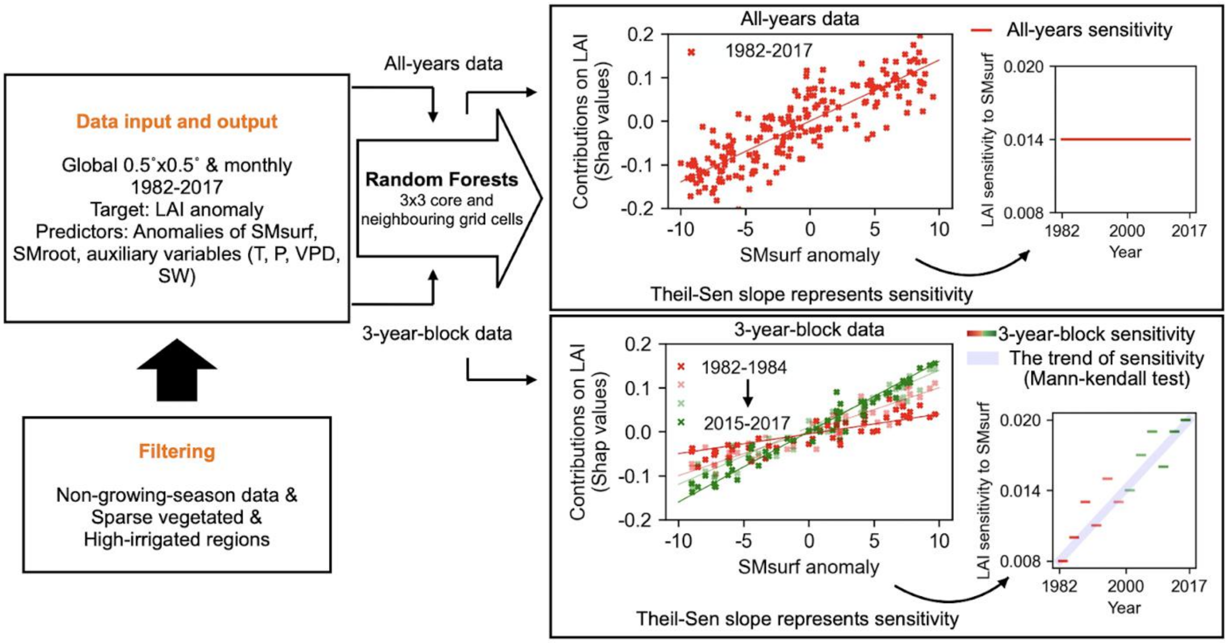

Supplementary Figure 1: The flow chat of data-processing and sensitivity analysis. Details can be found in Methods: Data-processing, Auxiliary data, Overall sensitivity and Sensitivity trends, respectively. Hydro-climate data anomalies are the predictor variables and LAI anomaly is the target variable. Random forests and SHAP values are used to isolated contributions of near-surface (or sub-surface) soil moisture to LAI for each 3x3 grid cells, and Theil-sen regression is used to estimate the linear sensitivity of LAI to near-surface (or sub-surface) soil moisture from all-years data and 3-year-block data, separately. To note that values of x and y axes in conceptual sub-figures do not have any real meaning. T denotes temperature, P denotes precipitation, VPD denotes vapor pressure deficit, and SW denotes incoming short-wave solar radiation.

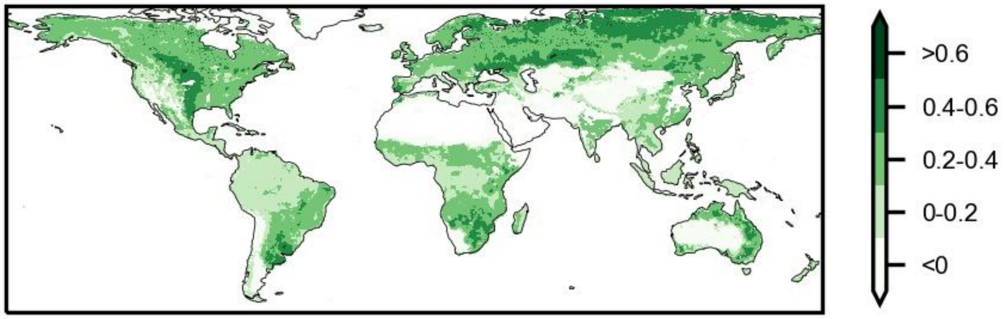

Supplementary Figure 2: The performance (out-of-bag R^2) of grid cell-based random forest models predicting monthly LAI from hydro-climate anomalies from ERA5-Land during 1982 to 2017. We display the mean R^2 across results from 5 observed LAI products.

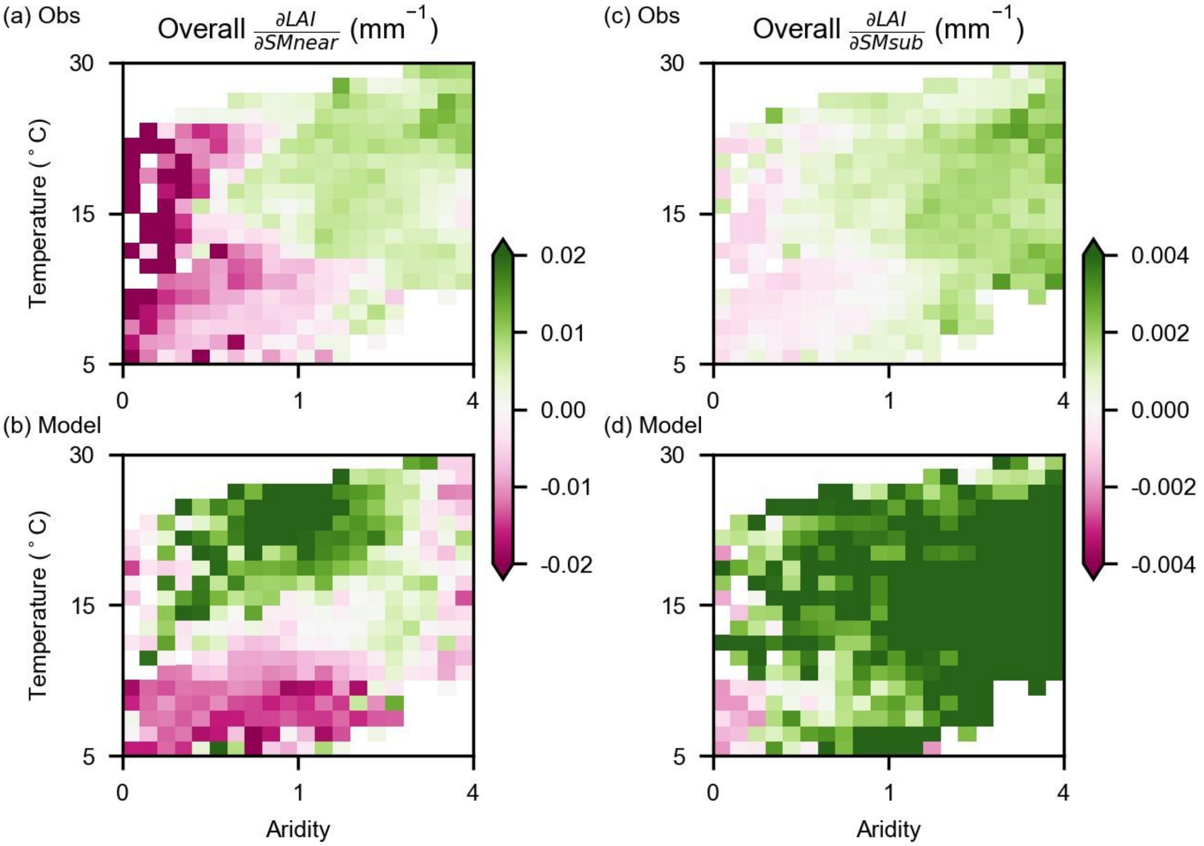

Supplementary Figure 3: Overall sensitivity of LAI to near-surface soil moisture (Overall $\frac{\partial LAI}{\partial SMnear}$) and sub-surface soil moisture (Overall $\frac{\partial LAI}{\partial SMsub}$) from observations (Obs) and LSMs (Model), given as respective ensemble means across climate regimes. Values higher than 1 in aridity index denote dry conditions (Methods: Auxiliary data).

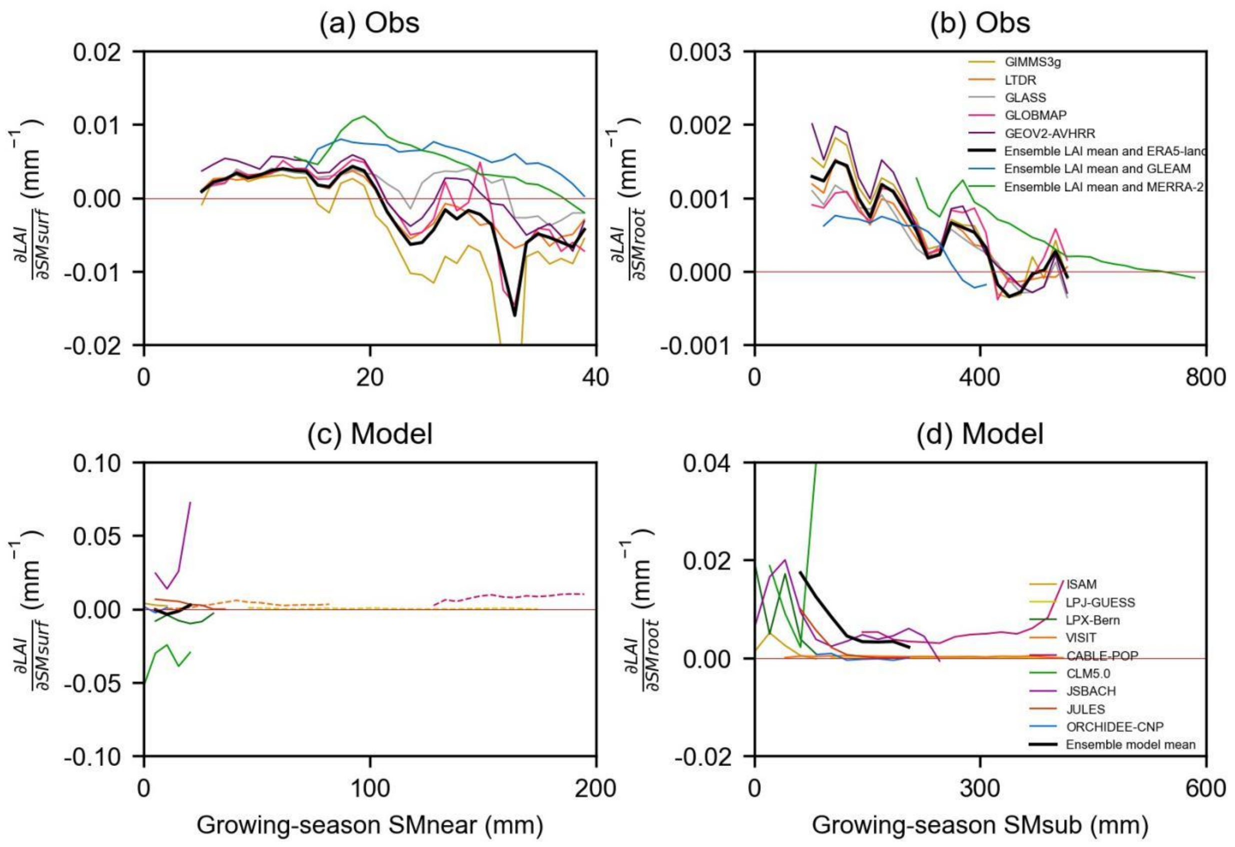

Supplementary Figure 4: As Fig. 2 but providing individual response functions of global LAI sensitivity to soil moisture from individual observational and LSMs results. (a) Response functions of LAI sensitivity ($\frac{\partial LAI}{\partial SMnear}$) to growing-season (see Methods: Data pre-processing) mean near-surface soil moisture (SMnear) from observations (Obs) across global grid cells. The black line shows the ensemble means and colored lines show observational results from individual products with visible labels. (b) Similar to (a) but for LAI sensitivity to sub-surface soil moisture ($\frac{\partial LAI}{\partial SMsub}$). (c) and (d) Similar to (a) and (b) but for LSMs results (Model). All panels only include global grid cells with significant LAI sensitivities to soil moisture. To infer significant LAI sensitivities, two-sided significance tests are done for each grid cell at the $p<0.01$ level as assessed with Theil-sen regressions. Single LSMs results not being accounted for averaged results (black lines) due to incompatible value ranges of near-surface soil moisture amounts are marked by dashed lines in (c).

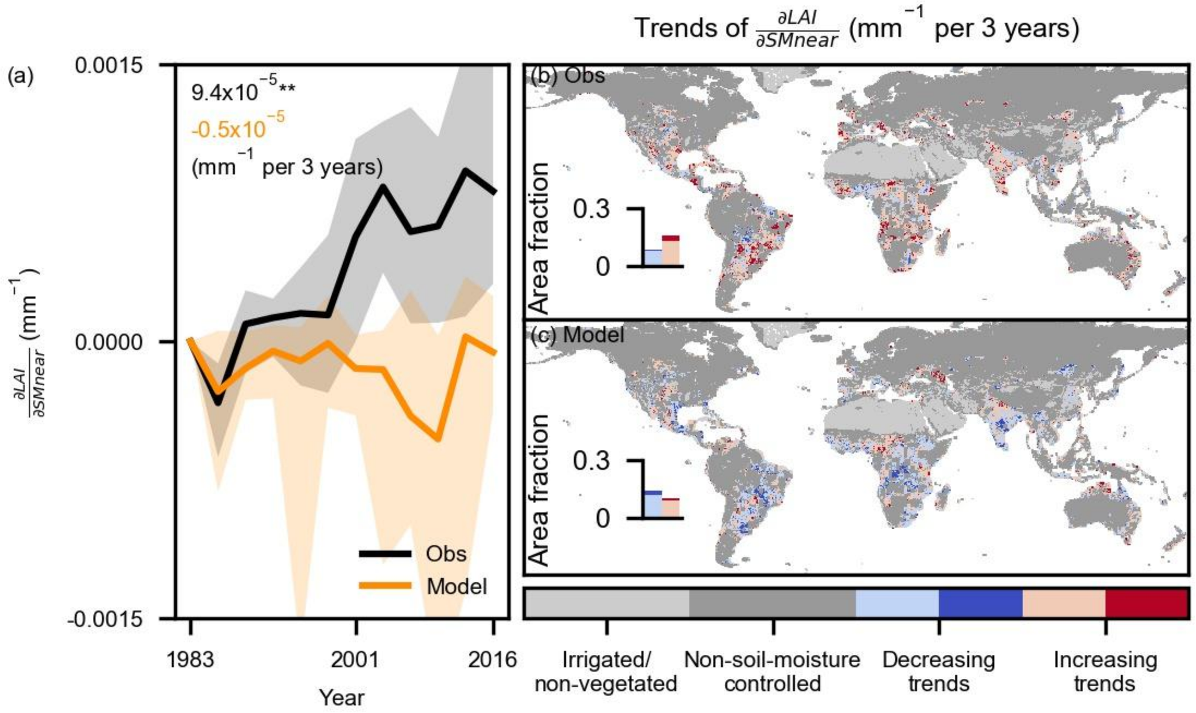

Supplementary Figure 5: Similar as in Fig. 3 but for trends in LAI sensitivity to near-surface soil moisture (SMnear). (a) Temporal variations of global mean LAI sensitivity to near-surface soil moisture ($\frac{\partial LAI}{\partial SMnear}$) are computed by 3-year blocks between 1982 and 2017. The y-axis denotes the change since 1982 in respective products or models. Solid lines denote the median results from ensemble observations (Obs) and LSMs (Model); Shaded areas denote interquartile ranges of LAI sensitivity from multiple LAI products and models; Text denotes slopes of trends; ** denotes passing the two-sided significance test as assessed with Mann-Kendall at $p<0.01$, otherwise $p>0.05$ (Methods: Sensitivity trends). (b) Trends of LAI sensitivity to sub-surface soil moisture ($Trends\ of\ \frac{\partial LAI}{\partial SMnear}$) in observations and models using their ensemble means. (c) Similar to (b) but for land surface models. Insets indicate the area fraction of decreasing and increasing trends within the study area. Light blue and red colors denote insignificant changes ($p>=0.1$); dark blue and red colors denote significant changes ($p<0.1$). In (b) and (c), two-sided significance tests are done for each grid cell at the $p<0.1$ level as assessed with Mann-Kendall's test.

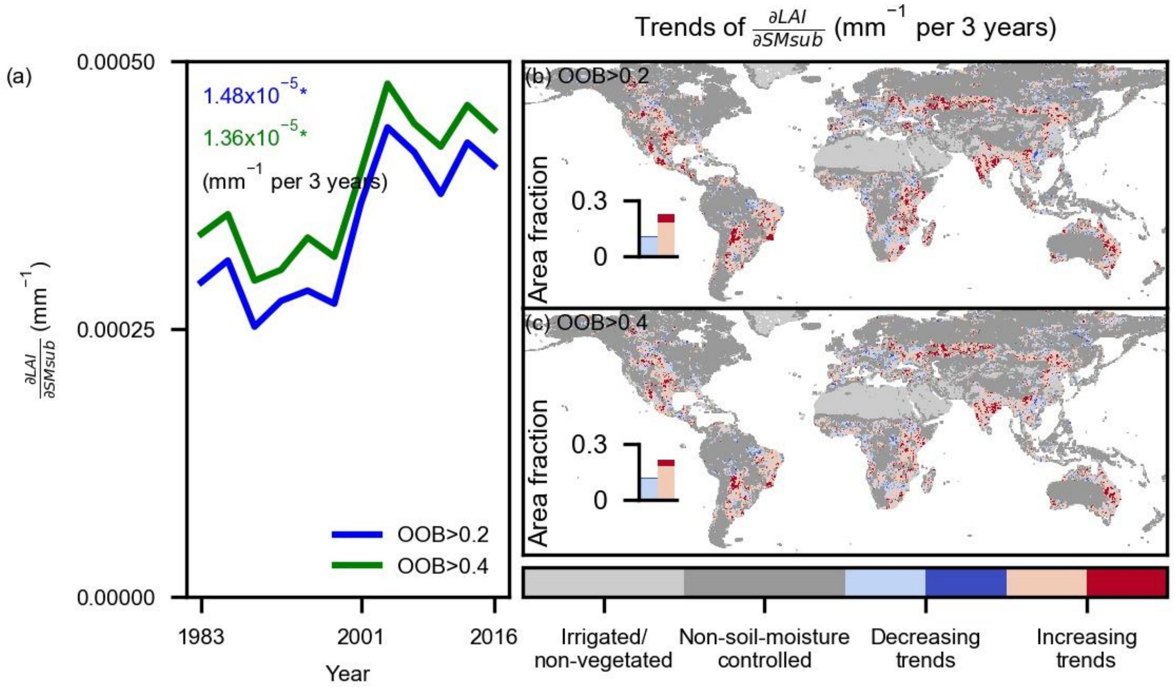

Supplementary Figure 6: Similar as in Fig. 3 but for overall and 3-year-block sensitivities derived using different thresholds of cross-validation out-of-bag R^2. (a) Temporal variations of global mean LAI sensitivity to sub-surface soil moisture ($\frac{\partial LAI}{\partial SMsub}$) as computed from 3-year blocks between 1982 and 2017. Numbers in the upper left corner denote slopes; * denotes passing the two-sided significance test as assessed with Mann-Kendall at $p<0.05$ (Methods: Sensitivity trends). (b, c) Trends of LAI sensitivity to sub-surface soil moisture (Trends of $\frac{\partial LAI}{\partial SMsub}$). Insets indicate the area fraction of decreasing and increasing trends within the study area. Light blue and red colors denote insignificant changes ($p>=0.1$); dark blue and red colors denote significant changes ($p<0.1$). In (b) and (c), two-sided significance tests are done for each grid cell at the $p<0.1$ level as assessed with Mann-Kendall's test.

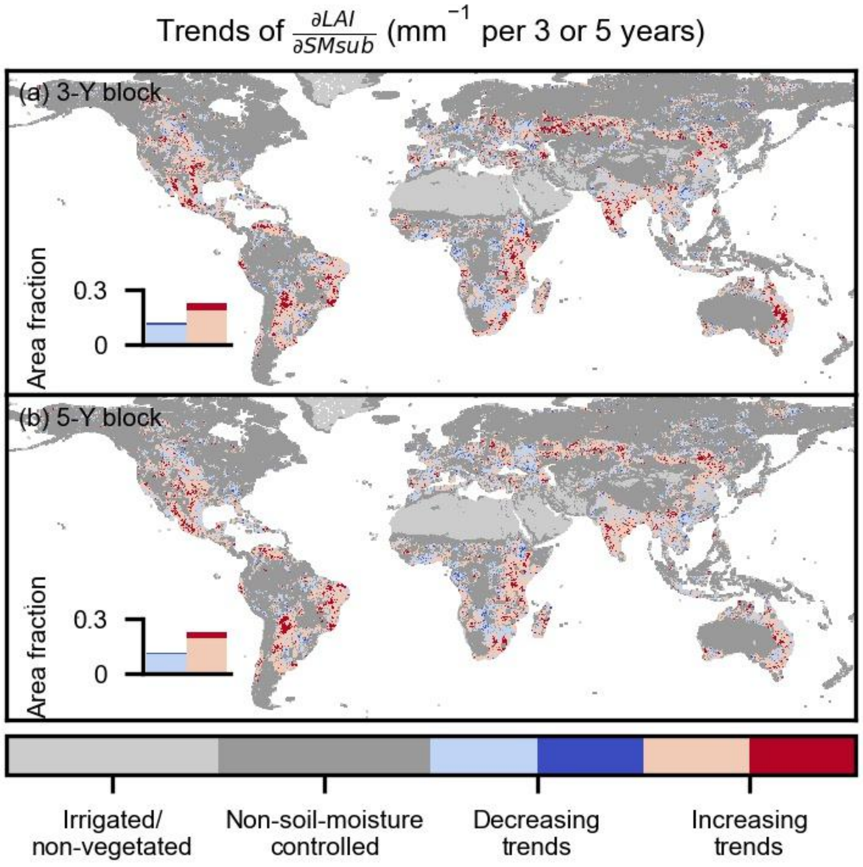

Supplementary Figure 7: Similar as in Fig. 3 but for global trends in observed LAI sensitivity to sub-surface soil moisture (Trends of $\frac{\partial LAI}{\partial SMsub}$) inferred by different subsets of data. (a) 3-Y blocks from 1982 to 2017 (1982-1984, 1985-1987, …, 2015-2017) and (b) 5-Y blocks from 1982 to 2016 (1982-1986, 1987-1991, …, 2012-2016). Insets indicate the area fraction of decreasing and increasing trends within the study area. Light blue and red colors denote insignificant changes ($p>=0.1$); dark blue and red colors denote significant changes ($p<0.1$). In (a) and (b), two-sided significance tests are done for each grid cell at the $p<0.1$ level as assessed with Mann-Kendall's test.

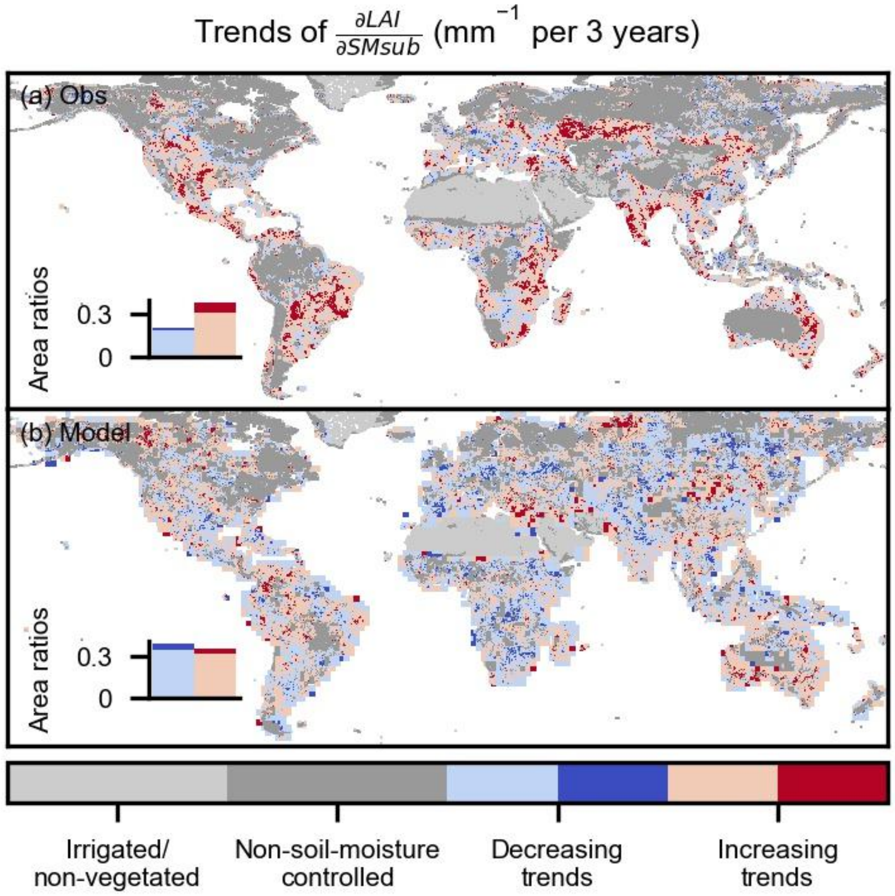

Supplementary Figure 8: As Fig. 3 but providing a full version of global trends in LAI sensitivity to sub-surface soil moisture (Trends of $\frac{\partial LAI}{\partial SMsub}$). (a) observed sensitivity trends (Obs) without additional filters by positive overall sensitivity inferred from LSMs. (b) sensitivity trends from LSMs (Model) without additional filters by positive overall sensitivity inferred from observational results. Insets indicate the area fraction of decreasing and increasing trends within the study area. Light blue and red colors denote insignificant changes ($p>=0.1$); dark blue and red colors denote significant changes ($p<0.1$). In (a) and (b), two-sided significance tests are done for each grid cell at the $p<0.1$ level as assessed with Mann-Kendall's test.

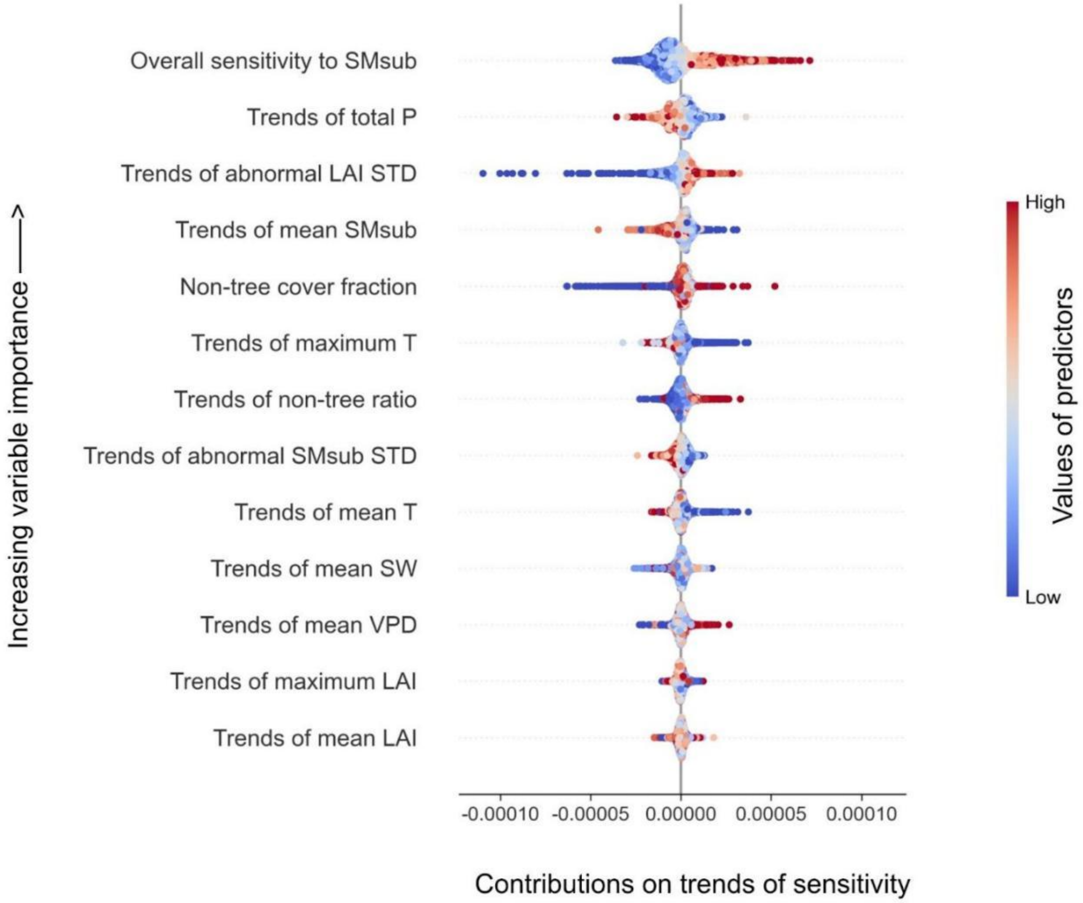

Supplementary Figure 9: Relative importance and marginal contributions (Shap values) of multiple factors to observed global trends in LAI sensitivity to sub-surface soil moisture (SMsub). STD denotes the standard deviation, T denotes temperature, P denotes precipitation, VPD denotes vapor pressure deficit, and SW denotes incoming short-wave solar radiation.

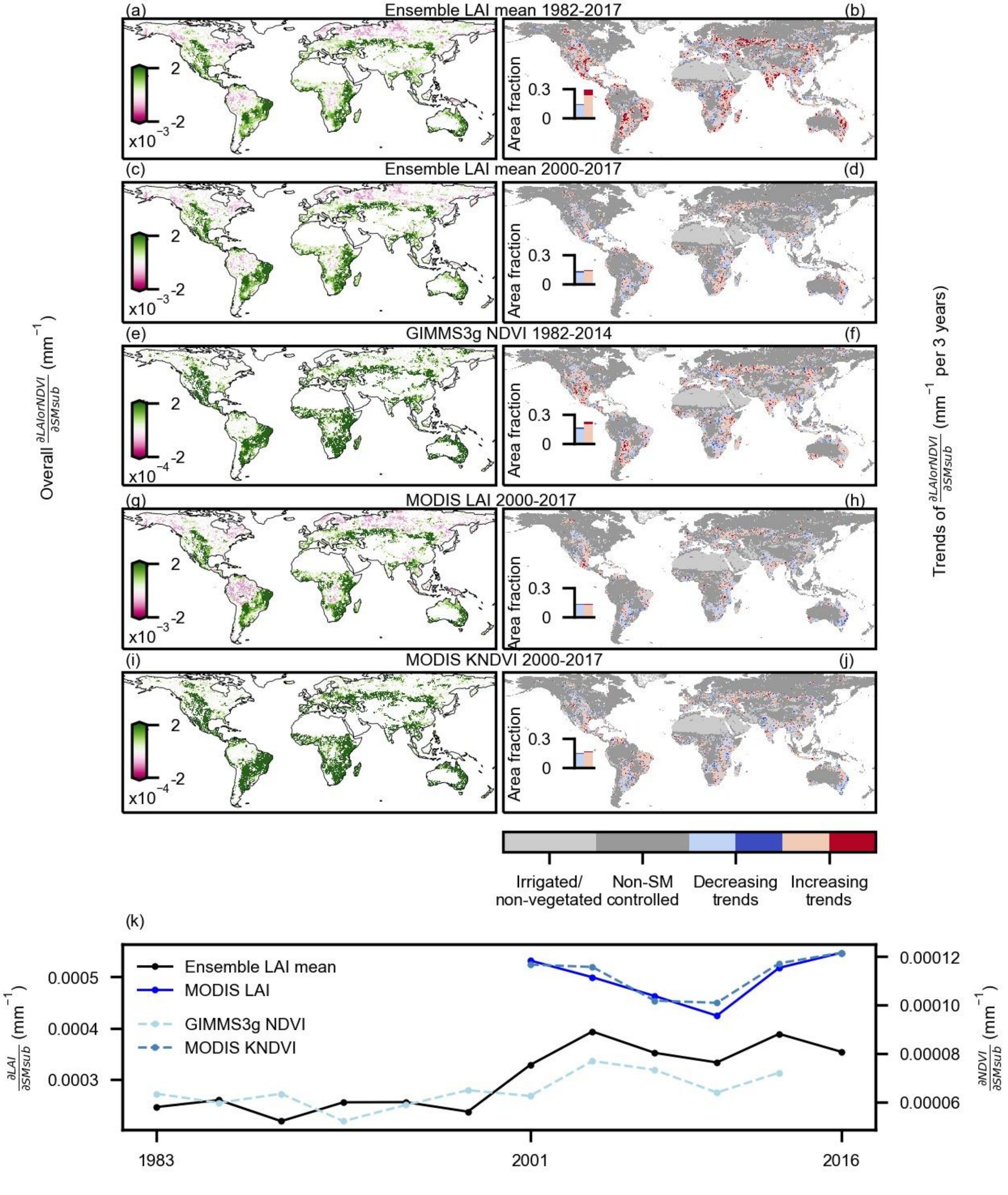

Supplementary Figure 10: Multiple-product comparisons of overall sensitivity, temporal variations of sensitivity and sensitivity trends of observed vegetation indices to sub-surface soil moisture. (a, c, e, h, i) Multiple-product comparisons of overall vegetation sensitivity to sub-surface soil moisture (Overall $\frac{\partial LAI\ or\ NDVI}{\partial SMsub}$). (a, c, e, h, i) apply the two-sided significance test

at the $p<0.01$ level as assessed with Theil-sen regressions for each grid cell, and grid cells which pass the significance test are colored by pink or green. (b, d, f, h, j) Multiple-product comparisons of global trends in vegetation sensitivity to sub-surface soil moisture (Trends of $\frac{\partial LAI\ or\ NDVI}{\partial SMsub}$). Insets indicate the area fraction of decreasing and increasing trends in the study area. Light blue and red colors denote insignificant changes ($p>=0.1$); dark blue and red colors denote significant changes ($p<0.1$). In (b, d, f, h, j), two-sided significance tests are done for each grid cell at the $p<0.1$ level as assessed with Mann-Kendall's test. (k) Multiple-product comparisons of temporal variations of LAI sensitivity to sub-surface soil moisture ($\frac{\partial LAI\ or\ NDVI}{\partial SMsub}$). Multiple greenness products are as follows: averaged LAI from 5 long-term products (Ensemble LAI mean), MODIS LAI, GIMMS3g NDVI, MODIS kernel NDVI (MODIS kNDVI).

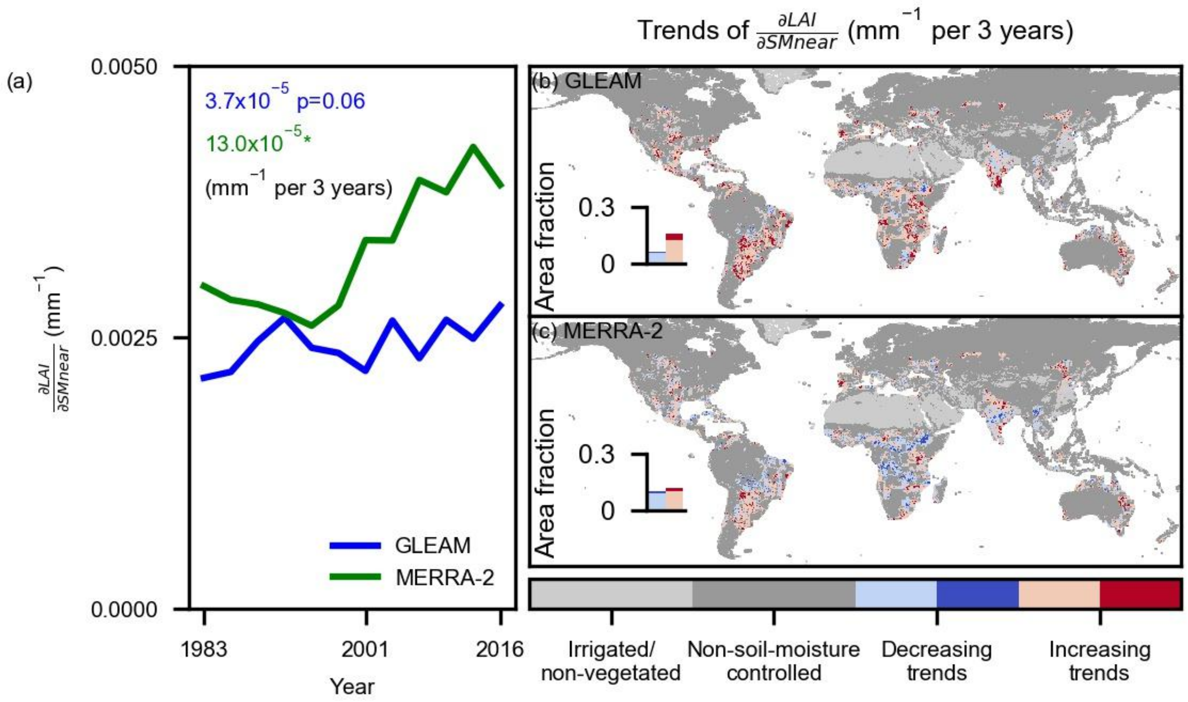

Supplementary Figure 11: Similar as in Fig. 3 but for trends in observed LAI sensitivity to near-surface soil moisture from Gleam and MERRA-2. (a) Temporal variations of global mean LAI sensitivity to near-surface soil moisture ($\frac{\partial LAI}{\partial SMnear}$) are computed by 3-year blocks between 1982 and 2017. Text denotes slopes of trends; * denotes passing a two-sided significance test as assessed with Mann-Kendall at $p<0.05$ (Methods: Sensitivity trends). (b, c) Trends of LAI sensitivity to sub-surface soil moisture from Gleam and MERRA-2. Insets indicate the area fraction of decreasing and increasing trends within the study area. Light blue and red colors denote insignificant changes ($p>=0.1$); dark blue and red colors denote significant changes ($p<0.1$). In (b) and (c), two-sided significance tests are done for each grid cell at the $p<0.1$ level as assessed with Mann-Kendall's test.

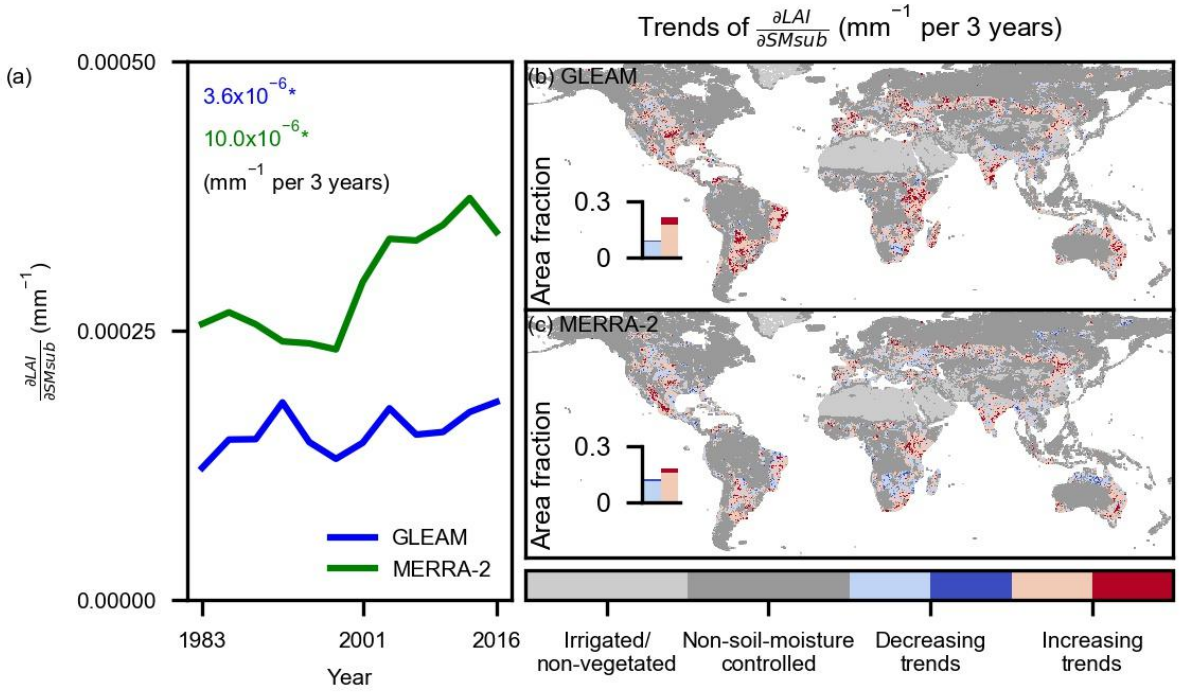

Supplementary Figure 12: Similar as in Fig. 3 but for trends in observed LAI sensitivity to sub-surface soil moisture from Gleam and MERRA-2. (a) Temporal variations of global mean LAI sensitivity to sub-surface soil moisture ($\frac{\partial LAI}{\partial SMsub}$) are computed by 3-year blocks between 1982 and 2017. Text denotes slopes of trends; * denotes passing a two-sided significance test as assessed with Mann-Kendall at $p<0.05$ (Methods: Sensitivity trends). (b, c) Trends of LAI sensitivity to sub-surface soil moisture from Gleam and MERRA-2. Insets indicate the area fraction of decreasing and increasing trends within the study area. Light blue and red colors denote insignificant changes ($p>=0.1$); dark blue and red colors denote significant changes ($p<0.1$). In (b) and (c), two-sided significance tests are done for each grid cell at the $p<0.1$ level as assessed with Mann-Kendall's test.

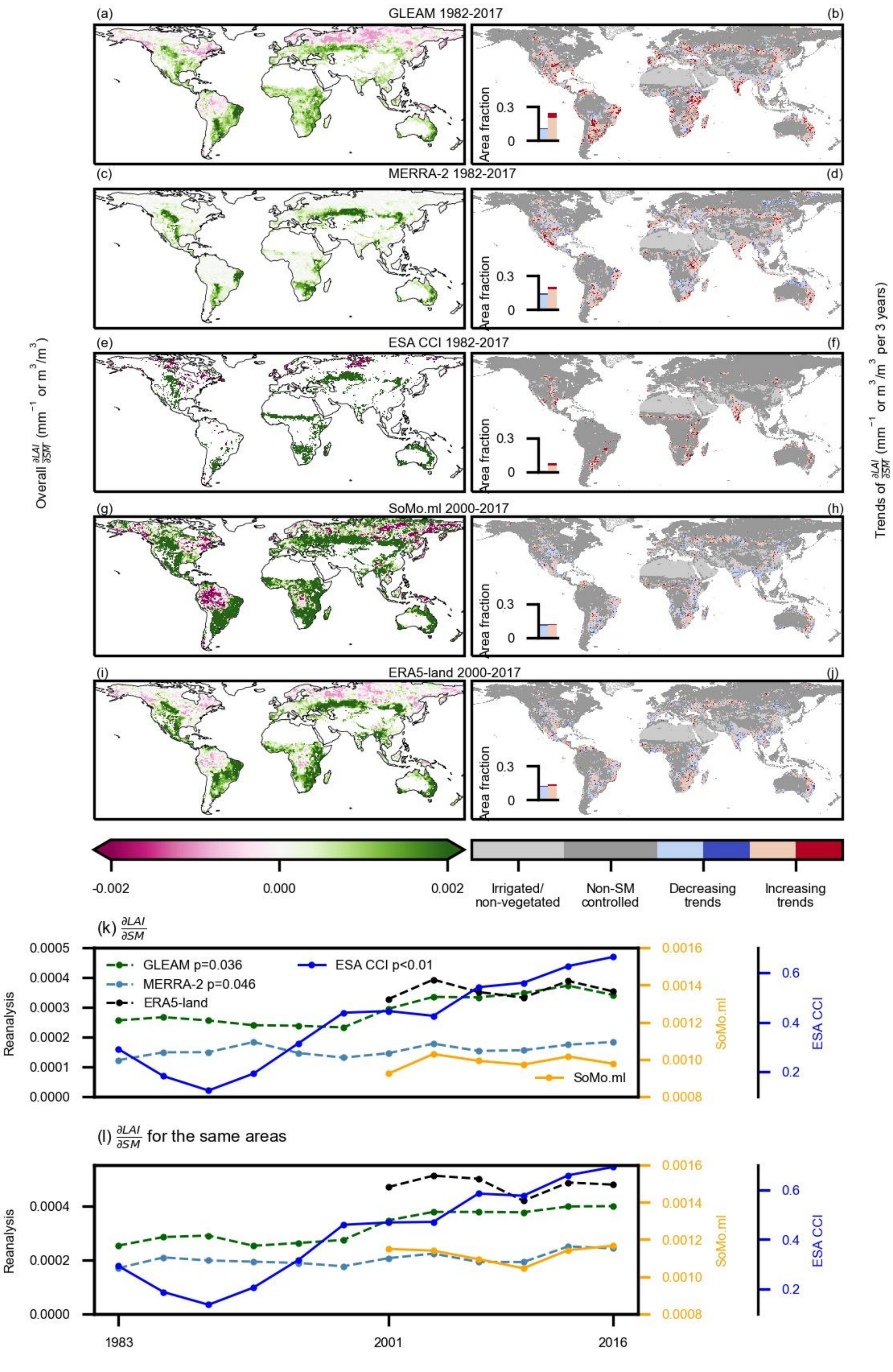
(a)
GLEAM 1982-2017
(b)
(c)
MERRA-2 1982-2017
(d)
(e)
ESA CCI 1982-2017
(f)
(g)
SoMo.ml 2000-2017
(h)
(i)
ERA5-land 2000-2017
(j)
Area fraction
0.3
0
Overall ∂LAI/∂SM (mm⁻¹ or m³/m³)
Trends of ∂LAI/∂SM (mm⁻¹ or m³/m³ per 3 years)
-0.002
0.000
0.002
Irrigated/ non-vegetated
Non-SM controlled
Decreasing trends
Increasing trends
(k) ∂LAI/∂SM
GLEAM p=0.036
MERRA-2 p=0.046
ERA5-land
ESA CCI p<0.01
SoMo.ml
Reanalysis
SoMo.ml
ESA CCI
(l) ∂LAI/∂SM for the same areas
1983
2001
2016

Supplementary Figure 13: Comparisons of overall sensitivity, temporal variations of sensitivity and sensitivity trends of LAI to multiple soil moisture (SM) products. Except for ESA CCI surface soil moisture, all the other products are sub-surface soil moisture. (a, c, e, h, i) Overall LAI sensitivity to soil moisture (Overall $\frac{\partial LAI}{\partial SM}$). (a, c, e, h, i) apply the two-sided significance test at the p<0.01 level as assessed with Theil-sen regressions for each grid cell, and grid cells which pass the significance test are colored by pink or green. (b, d, f, h, j) Global trends in LAI sensitivity to soil moisture (Trends of $\frac{\partial LAI}{\partial SM}$). Insets indicate the area fraction of decreasing and increasing trends in the study area. Light blue and red colors denote insignificant changes (p>=0.1); dark blue and red colors denote significant changes (p<0.1). In (b, d, f, h, j), two-sided significance tests are done for each grid cell at the p<0.1 level as assessed with Mann-Kendall's test. (k) Temporal variations of LAI sensitivity to soil moisture ($\frac{\partial LAI}{\partial SM}$) averaged from global grid cells and (l) averaged from the same global areas which are available from all soil moisture analyses. P values from the two-sided significance test are indicated in (k) as assessed with Mann-Kendall's test. ESA CCI near-surface soil moisture is expressed in m^3/m^3, while all other sub-surface soil moisture products are in mm.

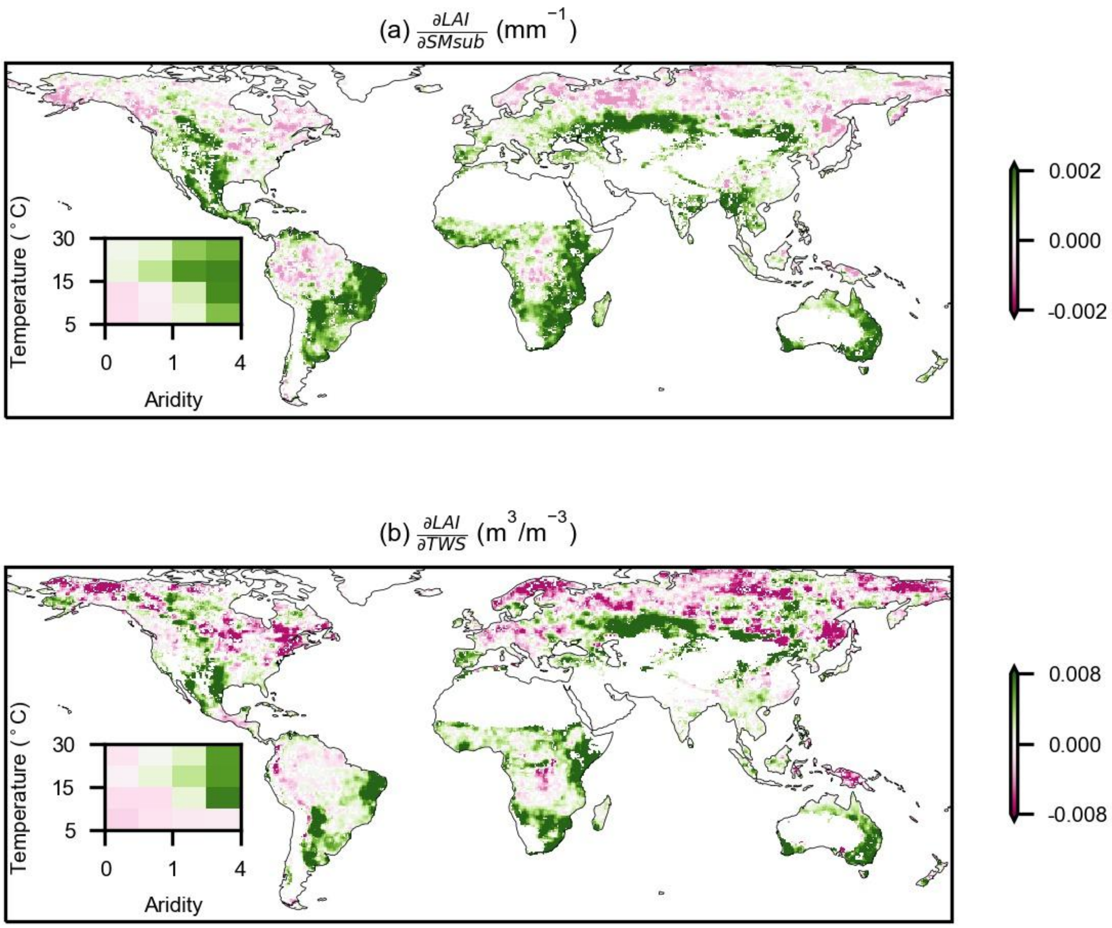

Supplementary Figure 14: Global sensitivity of LAI to (a) ERA5-Land sub-surface soil moisture ($\frac{\partial LAI}{\partial SMsub}$) and (b) GRACE total water storage ($\frac{\partial LAI}{\partial TWS}$) in the period 2003-2017. Inserts present median values of overall sensitivity across climate regimes. (a) and (b) apply the two-sided significance test at the p<0.01 level as assessed with Theil-sen regressions for each grid cell, and grid cells which pass the significance test are colored by pink or green.

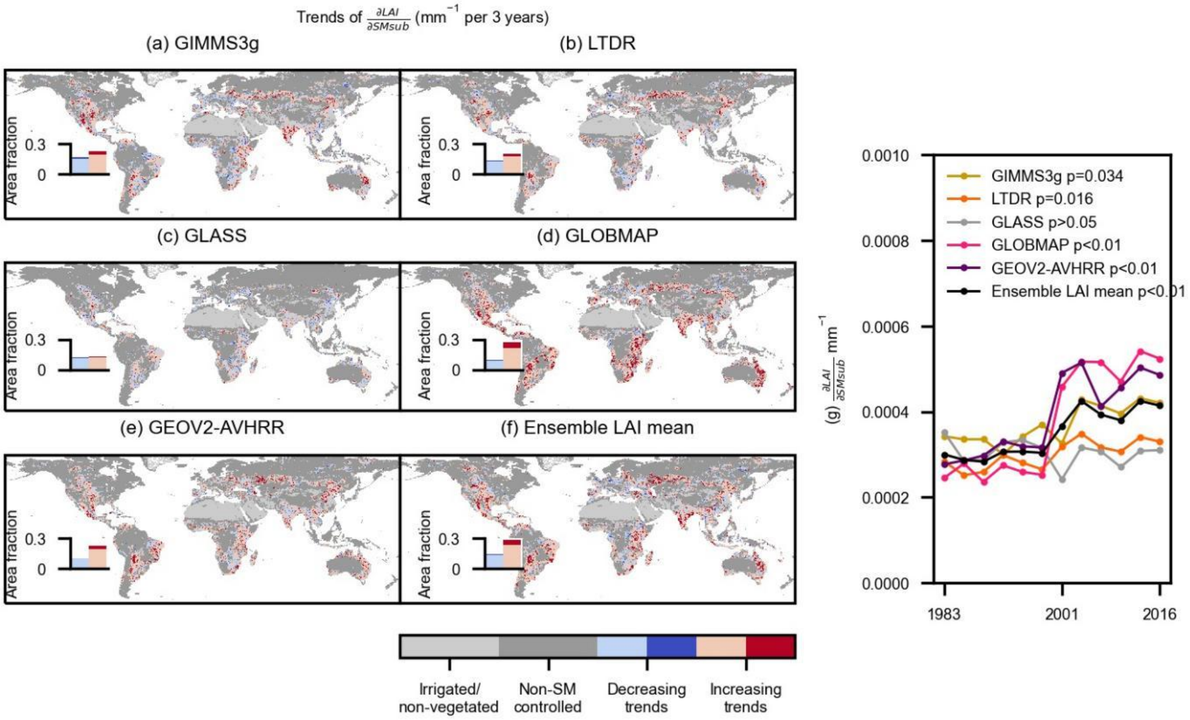

Supplementary Figure 15: Trends of LAI sensitivity and temporal variations of sensitivity to sub-surface soil moisture (SMsub) for individual LAI products. (a-f) Trends of LAI sensitivity to sub-surface soil moisture ($Trends\ of\ \frac{\partial LAI}{\partial SMsub}$). (g) Temporal variations of global LAI sensitivity to sub-surface soil moisture ($\frac{\partial LAI}{\partial SMsub}$). Trends of LAI sensitivity and temporal variations of LAI sensitivity to sub-surface soil moisture for individual LAI products. (a-f) Trends of LAI sensitivity to sub-surface soil moisture. (g) Temporal variations of global LAI sensitivity to sub-surface soil moisture. P values from the two-sided significance test are indicated in (g) as assessed with Mann-Kendall's test.
