# Supplementary material for: Widespread increasing vegetation sensitivity to soil moisture
Source: Nat Commun. 2022 Jul 8;13:3959. doi: 10.1038/s41467-022-31667-9 (PMC9270344; doi:10.1038/s41467-022-31667-9)
Supplement: Supplementary file 2 — Reporting Summary [file 41467_2022_31667_MOESM2_ESM.pdf]

nature portfolio

Corresponding author(s): Wantong Li

Last updated by author(s): Jun 20, 2022

Reporting Summary

Nature Portfolio wishes to improve the reproducibility of the work that we publish. This form provides structure for consistency and transparency in reporting. For further information on Nature Portfolio policies, see our Editorial Policies and the Editorial Policy Checklist.

Statistics

For all statistical analyses, confirm that the following items are present in the figure legend, table legend, main text, or Methods section.

n/a	Confirmed	
☐	☒	The exact sample size (*n*) for each experimental group/condition, given as a discrete number and unit of measurement
☐	☒	A statement on whether measurements were taken from distinct samples or whether the same sample was measured repeatedly
☐	☒	The statistical test(s) used AND whether they are one- or two-sided<br>*Only common tests should be described solely by name; describe more complex techniques in the Methods section.*
☐	☒	A description of all covariates tested
☐	☒	A description of any assumptions or corrections, such as tests of normality and adjustment for multiple comparisons
☐	☒	A full description of the statistical parameters including central tendency (e.g. means) or other basic estimates (e.g. regression coefficient) AND variation (e.g. standard deviation) or associated estimates of uncertainty (e.g. confidence intervals)
☐	☒	For null hypothesis testing, the test statistic (e.g. *F*, *t*, *r*) with confidence intervals, effect sizes, degrees of freedom and *P* value noted<br>*Give P values as exact values whenever suitable.*
☒	☐	For Bayesian analysis, information on the choice of priors and Markov chain Monte Carlo settings
☒	☐	For hierarchical and complex designs, identification of the appropriate level for tests and full reporting of outcomes
☒	☐	Estimates of effect sizes (e.g. Cohen's *d*, Pearson's *r*), indicating how they were calculated

Our web collection on statistics for biologists contains articles on many of the points above.

Software and code

Policy information about availability of computer code

Data collection	N/A
Data analysis	Python 3.8.12; scikit-learn 1.0.1; shap 0.40.0

For manuscripts utilizing custom algorithms or software that are central to the research but not yet described in published literature, software must be made available to editors and reviewers. We strongly encourage code deposition in a community repository (e.g. GitHub). See the Nature Portfolio guidelines for submitting code & software for further information.

Data

Policy information about availability of data

All manuscripts must include a data availability statement. This statement should provide the following information, where applicable:

- Accession codes, unique identifiers, or web links for publicly available datasets
- A description of any restrictions on data availability
- For clinical datasets or third party data, please ensure that the statement adheres to our policy

Data availability:
Five long-term LAI products GIMMS3g V1, LTDR V5, GLASS V40, GLOBMAP V3 and GEOV2-AVHRR are available at http://sites.bu.edu/cliveg/datacodes/, https://ladsweb.modaps.eosdis.nasa.gov/, http://www.glass.umd.edu/, https://zenodo.org/record/4700264#.YRPUpNMzZlc, and https://www.theia-land.fr/en/geov2-avhrr-monitoring-changes-in-vegetation-on-a-global-scale-over-the-last-38-years/, respectively. GEOV2-AVHRR was produced and distributed by CNES based on the

March 2021

algorithm developed by CREAF and INRAE in the framework of the Theia Land Data Centre. GIMMS3g v1 NDVI, MOD15A2H LAI and MOD13C2 NDVI are available at https://lpdaac.usgs.gov/products. ERA5-Land climate and soil moisture reanalysis datasets are available at https://www.ecmwf.int/en/era5-land. GLEAM soil moisture reanalysis is from https://www.gleam.eu/. MERRA-2 soil moisture reanalysis is from https://gmao.gsfc.nasa.gov/reanalysis/MERRA-2/FAQ/. Fractional vegetation cover is from the AVHRR vegetation continuous fields products (VCF5KYR, https://lpdaac.usgs.gov/products/vcf5kyrv001/). Simulations from TRENDY land surface models are available on request to S.S. (s.a.sitch@exeter.ac.uk) and P.F. (p.friedlingstein@exeter.ac.uk)..
All data used in this article are publicly available and listed in the supplementary materials.

Human research participants

Policy information about studies involving human research participants and Sex and Gender in Research.

Reporting on sex and gender	N/A
Population characteristics	N/A
Recruitment	N/A
Ethics oversight	N/A

Note that full information on the approval of the study protocol must also be provided in the manuscript.

Field-specific reporting

Please select the one below that is the best fit for your research. If you are not sure, read the appropriate sections before making your selection.

☐ Life sciences ☐ Behavioural & social sciences ☒ Ecological, evolutionary & environmental sciences

For a reference copy of the document with all sections, see nature.com/documents/nr-reporting-summary-flat.pdf

Ecological, evolutionary & environmental sciences study design

All studies must disclose on these points even when the disclosure is negative.

Study description	We assess the response of global vegetation to short-term soil moisture changes, and infer related trends between 1982 – 2017 using an explainable machine learning approach.
Research sample	We use Earth observations to analyze several long-term records of global vegetation indices reflecting vegetation greenness and productivity, in conjunction with multiple soil moisture reanalyses covering the near-surface and sub-surface layers and climate reanalyses from 1982 to 2017. We also use variables mentioned above from land surface models from Trendy v7.
Sampling strategy	We collect all predictors and target data during 1982-2017 from one grid cell and the surrounding grid cells (3x3 shape) to train a random forest model for the core grid cell if more than 50 data points are included. Random forests have the advantages of bootstrap aggregating and non-distribution assumption.
Data collection	No sampling data are originally collected by the authors; The authors download the data from public websites and list the public links in the manuscript. The associated instruments to collect the data are also indicated in corresponding websites; Wantong Li and Ulrich Weber download the all data used in the analyses using high performance computers in Department of Biogeochemical Integration, Max Planck Institute for Biogeochemistry, Germany.
Timing and spatial scale	Since satellite-observed LAI is available from 1982 until now and land surface models provide LAI data until 2017 at the monthly time scale, we analyze the global vegetation and hydro-climate data from 1982 to 2017 at monthly time-scale for all observational and modeled LAI and soil moisture data.
Data exclusions	No data were excluded from the analyses.
Reproducibility	All attempts to repeat the experiment were successful.
Randomization	Sampling data to train models are randomly selected using random forest algorithms.
Blinding	Sampling data to train models are randomly selected using random forest algorithms.

Did the study involve field work? ☐ Yes ☒ No

Reporting for specific materials, systems and methods

We require information from authors about some types of materials, experimental systems and methods used in many studies. Here, indicate whether each material, system or method listed is relevant to your study. If you are not sure if a list item applies to your research, read the appropriate section before selecting a response.

Materials & experimental systems

n/a	Involved in the study
☒	☐ Antibodies
☒	☐ Eukaryotic cell lines
☒	☐ Palaeontology and archaeology
☒	☐ Animals and other organisms
☒	☐ Clinical data
☒	☐ Dual use research of concern

Methods

n/a	Involved in the study
☒	☐ ChIP-seq
☒	☐ Flow cytometry
☒	☐ MRI-based neuroimaging
